# Understanding image-text relations and news values for multimodal news analysis

**DOI:** 10.3389/frai.2023.1125533

**Published:** 2023-05-02

**Authors:** Gullal S. Cheema, Sherzod Hakimov, Eric Müller-Budack, Christian Otto, John A. Bateman, Ralph Ewerth

**Affiliations:** ^1^TIB – Leibniz Information Centre for Science and Technology, Hannover, Germany; ^2^Computational Linguistics, University of Potsdam, Potsdam, Germany; ^3^L3S Research Center, Leibniz University Hannover, Hannover, Germany; ^4^Department of English and Linguistics, Universität Bremen, Bremen, Germany

**Keywords:** multimodality, news analysis, news values, computational analytics, machine learning, image-text relations, semiotics, journalism

## Abstract

The analysis of news dissemination is of utmost importance since the credibility of information and the identification of disinformation and misinformation affect society as a whole. Given the large amounts of news data published daily on the Web, the empirical analysis of news with regard to research questions and the detection of problematic news content on the Web require computational methods that work at scale. Today's online news are typically disseminated in a multimodal form, including various presentation modalities such as text, image, audio, and video. Recent developments in multimodal machine learning now make it possible to capture basic “descriptive” relations between modalities–such as correspondences between words and phrases, on the one hand, and corresponding visual depictions of the verbally expressed information on the other. Although such advances have enabled tremendous progress in tasks like image captioning, text-to-image generation and visual question answering, in domains such as news dissemination, there is a need to go further. In this paper, we introduce a novel framework for the computational analysis of multimodal news. We motivate a set of more complex image-text relations as well as multimodal news values based on real examples of news reports and consider their realization by computational approaches. To this end, we provide (a) an overview of existing literature from *semiotics* where detailed proposals have been made for taxonomies covering diverse image-text relations generalisable to any domain; (b) an overview of computational work that derives models of image-text relations from data; and (c) an overview of a particular class of news-centric attributes developed in journalism studies called news values. The result is a novel framework for multimodal news analysis that closes existing gaps in previous work while maintaining and combining the strengths of those accounts. We assess and discuss the elements of the framework with real-world examples and use cases, setting out research directions at the intersection of multimodal learning, multimodal analytics and computational social sciences that can benefit from our approach.

## 1. Introduction

News media today convey information about events worldwide in a broad variety of formats, including print, television, news websites, and social media platforms. News websites have evolved to use a broad range of presentation modalities, including text, photos, diagrams, and videos. With an unprecedented increase of information and ease of access and consumption due to the Internet, it has become increasingly important to analyse news content (Karlsson and Sjøvaag, 2016), evaluate its correctness, and understand the spread of information, regardless of the presentation modalities involved. Given the vast quantity of news articles, however, such analysis cannot be accomplished without computational methods, be that for empirical analysis of multimodal news for research or as a response to the highly-pressing need for (software) tools that help to identify and filter problematic news content on the Web and in social media.

**Figure 1:**
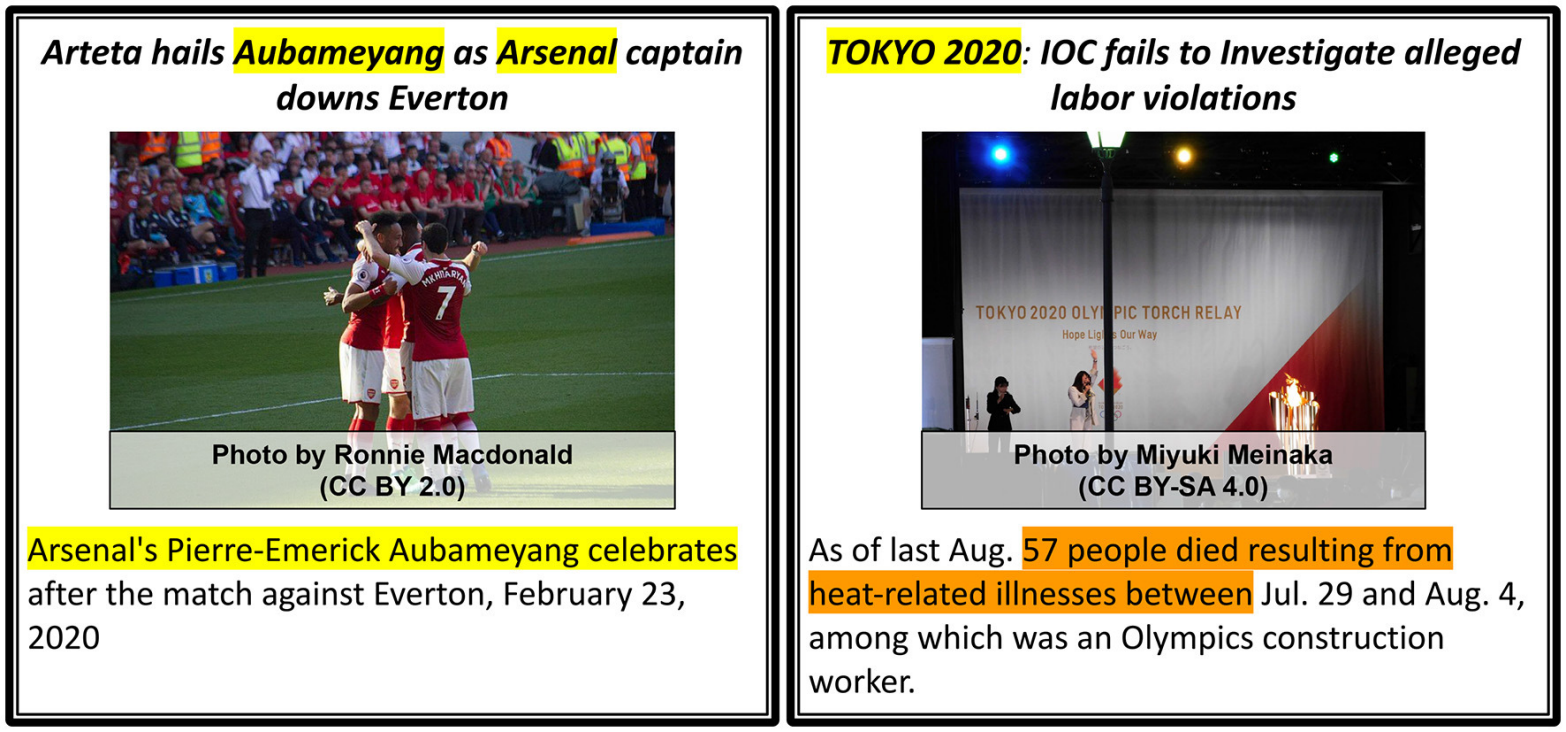
Examples of image-text pairs from news. **Left**: An additive relationship between image and text. **Right**: A stock photograph uncorrelated or contradictory to the text. Highlighted text shows the contradicting and overlapping aspects within the images respectively. In general, an image can have relations to text in different parts of an article, but here we focus on the headline (bold) and caption of the image as these already exhibit some of the complexity involved.

Multimodal news analysis involves several challenges, as shown in Figure 1. There, the two pairs contain different cross-modal relations, which add complexity to the conveyed message. The samples are both taken from sports news but exhibit different relations between image and text. The sample on the right shows a stock photograph of the *Tokyo Olympics 2020*. But this image is either uncorrelated or contradictory to the surrounding text because the text mentions an investigation resulting from the death of workers at Olympics construction sites. The image, in contrast, simply depicts two persons on stage celebrating Olympics torch relay. The information in both modalities does not convey a similar message and, indeed, taken at face value, may well suggest unwarranted connections – for example: are the people shown those failing to investigate? The sample on the left of the figure is very different in that the caption and image, in this case, are congruent. They exhibit a complementary and additive relation because the caption provides more information (opponent team and date) to that of the image. At the same time, the image complements the text by showing the mentioned football player.

These examples highlight the difficulty of analysing multimodal news articles where information from different modalities may stand in a broad range of interrelationships. Current computational approaches for news analysis mostly focus on *unimodal* and *data-driven* aspects like sentiment analysis (Godbole et al., 2007; Taj et al., 2019), stance detection (Hanselowski et al., 2018), and focus location estimation (D'Ignazio et al., 2014; Imani et al., 2017). Moreover, hardly any computational work has adopted relevant taxonomies of text-image relationships developed within semiotics (Barthes, 1977; Marsh and White, 2003; Martinec and Salway, 2005) or media studies (Bednarek, 2016; Caple et al., 2020), sometimes specifically for analyzing news articles. As a result, computational methods do not model cross-modal relations between text and images and cannot provide interpretations of the (overall) multimodal message, which can be caused by complex mechanisms known under the notion of meaning multiplication (Lemke, 1998; Bateman, 2014). In addition, the text-image relations described in the literature require closer connections with other aspects of news analysis. In this context, news values (Caple and Bednarek, 2016; Harcup and O'Neill, 2017) can play crucial roles in modulating the text-image relations that apply. News values are defined as aspects of events that make them “newsworthy”, such as the involvement of elite personalities (e.g., celebrities, politicians) or the impact of an event and its negative consequences. Bednarek and Caple (2017) suggest that the concept of news values can equally well be applied to text and image content, which needs to be considered from the perspective of automatic analysis, particularly with respect to interactions with text-image relations.

In this paper, therefore, we introduce a framework for the scalable analysis of multimodal news articles that covers a set of *computable* image-text relations and news values. First, we adapt existing models and taxonomies for image-text relations (Otto et al., 2019b) and expand them for multimodal news analysis, adding further semantic relations inspired by semiotics. Second, we adopt news values (Bednarek and Caple, 2017) from journalism studies, modify them regarding specific *multimodal* aspects, and add distinctive sub-categories. This set of defined relations and news values aims at enabling computational methods to provide more interpretable characterizations of articles, thus allowing for empirical analysis of multimodal news articles at scale. We also develop two additional aspects that respond to the two main stakeholder groups in the news process: publishers and readers. Therefore, we integrate *author intent* in our framework to capture the primary purpose of the news piece and incorporate *subjective interpretation* to allow for variation with respect to image-text relations and news values based on the target audience's background, experience, and demographics.

Overall, the framework presented in this paper covers interdisciplinary topics and contributes to research in multimodal analytics, machine learning, communication sciences and media studies. The principal contributions of this paper can be summarized as follows:

- We review and discuss a broad range of literature from semiotics, computational science, and journalism studies concerning news factors and consider methods for combining them.
- We develop a framework based on multimodal news content, author intent, news values, and subjective interpretation that addresses the entire process of news communication from production to consumption.
- We critically compare the taxonomy we develop with previously proposed taxonomies, and provide a large number of real-world news examples to motivate and explain different parts of the framework.
- Finally, we discuss applications and tools that can benefit from the framework. Hopefully, this will inspire new interdisciplinary research directions.

The remainder of the paper is structured as follows. In Section 2, we describe the related work from communication and computational science with respect to image-text relations to provide the context for our framework and identify research gaps. In Section 3, we explain our proposed framework with example use cases and discuss their computational aspects and implications. And lastly, in Section 4, we assess the framework in terms of applications and use-cases before concluding the paper with some directions for future research.

## 2. Related work and background

A wide variety of literature exists in multiple fields that have explored the relationship between different modalities and analyzed news from popular media outlets and social media. For current purposes, however, we focus in this section on relevant work for image and text relations in communication science and computational science, as these are equally important for understanding the content and importance of our proposals below. We briefly describe relevant previous work and provide a detailed review of that work motivating our own. In the case of computational approaches, we focus for the present paper on proposed taxonomies and applications of computational methods built using various machine learning models for analysing different forms of digital media.

### 2.1. Communication science

We first describe those approaches that can be generally applied to image-text pairs regardless of the domain and then discuss news values central to analysing and understanding news articles. The discussion follows a chronological order signifying the evolution of treatments of image-text relations over the years.

#### 2.1.1. Image-text relations

***Early and main taxonomies:*** In the pioneering work of Barthes (e.g., Barthes, 1977), three relations are introduced to capture the relative importance of a modality in an image-text pair and to characterize the functions (purposes) served by each modality. These relations are: (1) *Anchorage*: text supporting image in a way that the text guides the viewer in describing and interpreting an image, restricting the commonly assumed “polysemy” of the image; (2) *Illustration*: an image supporting text such that the image recasts in a pictorial form information largely already present in the text; and (3) *Relay*: where text and image exhibit a bidirectional and equal relationship such as complementarity or interdependence. Many semioticians have subsequently explored the integration of meaning in visual and verbal representation in specific domains, although without always specifying a system of relations such as that of Barthes. Most notable here are a study of verbal-visual relations in film documentaries by van Leeuwen (1991), a study of relating abstract images and text segments by Martin (1994), an analysis of scientific articles that combine tables, diagrams and text by Lemke (1998), and detailed analyses of inter-semiotic relations between images and text in advertisements by Stöckl (1997) and Royce (1998). A detailed overview of these and several other approaches discussed below is given in Bateman (2014).

Some later works combine Barthes' taxonomy with notable input from linguistics, in particular by adopting accounts of semantic relationships between clauses in grammar (Halliday, 1985; Halliday et al., 2014) as models for mapping image-text pairs into meaningful relations as well. Martinec and Salway (2005), for example, propose a taxonomy drawing on both Barthes and Halliday that characterizes relations by systematically distinguishing relative importance (status) and semantic relations (logico-semantic). Unique relations are then formed by cross-classifying along the two dimensions to provide detailed classes of image-text pairs combining *status* and *logico-semantic* information. For instance, they divide *equal status* further into *complementary* and *independent* to distinguish when both modalities are necessary to convey the message or when they contain overlapping information, respectively. Under semantic relations, they re-purpose Halliday's main types of *elaboration, extension* and *enhancement* to relate images and texts more generally, adding new information and enrichment of certain attributes (like time or place). In a related approach developed in the educational context, Unsworth (2007) also extends Martinec and Salway (2005) *logico-semantic* relations specifically for science textbooks but with different labeling of some relations suited for textbooks, articulating them further for more fine-grained dimensions or sub-categories. For instance, they add a *clarification* relation under *elaboration* suited to diagrams in textbooks, where a diagram provides clarification for the surrounding text. Some other works by Wunderli (1995) and van Leeuwen (2005) also specify cases of negative relations such as *contradiction* between image and text, e.g., by creating a contrast to draw attention to certain aspects or elements in each modality.

***Other taxonomies:*** Another relevant approach drawing on quite different considerations from communication studies is that of Marsh and White (2003). These authors develop contextual ties between image and text to improve information retrieval and document design. By analyzing articles in several subject areas, they identified 49 relationships grouped into three higher-level categories according to the closeness of the conceptual relationship between image and text. The three main categories are centered around the idea of an image as an *illustration*, and how this illustration relates to the surrounding text. Their categories are: (1) *Minimal*: illustration expressing little relation to the text; (2) *Close*: illustration expressing a close (highly related) relation to the text; and (3) *Transcendental*: illustration that is closely related, but also going beyond the text.

***Use-case studies:*** Several use-case studies have manually applied taxonomies of text-image relation to real-world problems and data analysis. Mehmet et al. (2014) work on social media semantics applies Unsworth's taxonomy to understand how messages and online conversations construct and convey meaning. Interestingly, the image-text pairs in this work are not necessarily co-present within single units but can exist on different platforms at different locations and times. In addition, both Wu (2014) and Nhat and Pha (2019) apply Unsworth's taxonomy to picture books and English comics for children, respectively. Several broader annotation-based studies then present results in terms of quantitative distributions of the relations found in selected texts (Moya Guijarro, 2014; Nhat and Pha, 2019).

#### 2.1.2. News values

***Seminal works and different perspectives:*** The systematic consideration of news values is generally traced back to the seminal study of Galtung and Ruge (1965) on Scandinavian news discourse. These authors introduce a list of news values divided into two broad categories: culture-free and culture-bound. Culture-free news values are those that are based solely on perception, such as *frequency* and *threshold* (e.g., a large or impactful event), whereas culture-bound news values involve judgements specific to a target culture, such as the presence of *elite persons or nations* and *negativity*, i.e., an event judged negatively by the target culture. Since then, several attempts have been made at redefining and extending these categories with more news values from different perspectives (Bell, 1991; Harcup and O'Neill, 2001; Brighton and Foy, 2007). For an event to be constructed as news, then, several factors are at play reflecting various aspects of the news production process. Caple and Bednarek (2016) usefully differentiate these factors into three categories as follows:

- **News writing objectives:** general goals associated with news writing, such as *clarity of expression, brevity, color, accuracy* and so on.
- **Selection factors:** any factors or criteria impacting whether or not a story becomes published that are not intrinsic to the presented news item itself, e.g., *commercial pressures, availability of reporters, deadlines* and so on.
- **News values:** the “newsworthy” aspects of actors, happenings and issues as established by a set of recognized values such as *negativity, proximity, timeliness* and so on.

Some researchers consider factors in news writing objectives and selection factors to be part of news values as well, but this blurs the line differentiating them from news values that are event-dependent and audience-centric. Furthermore, news values themselves can be produced from different perspectives defined succinctly by Bednarek and Caple (2012) and Caple and Bednarek (2016) as below:

- **Material perspective:** News values that exist in the actual events and people who are reported on in the news, that is, in events in their material reality.
- **Cognitive perspective:** News values that exist in the minds of journalists.
- **Discursive perspective:** News values that are constructed in the discourses involved in the production of news using language and image.

Although many researchers define news values from a *cognitive* perspective, Caple (2013) takes a *discursive* perspective, stating that the other process is highly subjective and may lead to a different interpretation because of every journalist's or news worker's assumptions about news values. For our work and analysis of multimodal news articles, we are then interested particularly in the discursive event-dependent approach (Caple and Bednarek, 2016) . The discursive approach gives a view of how news values are constructed from different modalities and provides crucial insights into how news media package events as “news”. From this discursive perspective, Bednarek and Caple (2012) define nine news values present in both language and image, which are *Negativity, Proximity, Timeliness, Prominence, Novelty, Consonance, Impact, Superlativeness* and *Aesthetics*. These news values and relevant changes in our framework are discussed in detail in Section 3.3.

***Use-case studies:*** While these news values are particularly defined for news stories from media channels, they also apply to social media news stories shared across platforms such as Facebook (Bednarek, 2016). Some other works consequently apply news value theory to news articles shared on Facebook (Park and Kaye, 2021), Twitter (Araujo and van der Meer, 2020) and the Russian social media site Vkontakte (Judina and Platonov, 2019). Park and Kaye (2021) studied the correlation between news values of social significance and deviance with social media users' tendency to like, comment and share mainstream news stories on Facebook. Araujo and van der Meer (2020) conducted a large study on 1.8 million tweets and showed that the news values of social impact, geographical closeness and facticity, among other factors, can explain the intensity of online activities. Harcup and O'Neill (2017) also investigate news values and adapt them for both actual news stories and news on social media by adding values like *shareability, follow-up*, and *entertainment*. Tandoc Jr et al. (2021) studied the “newsness” of fake news by examining fake articles based on news values, topic and format. Interestingly, this study found that most fake news articles included the news values of timeliness, negativity and prominence. The only difference between real and fake news that was found concerned objectivity: fake news tended to include the author's personal opinion (i.e., not objective) through adjectives and judgements not attributed to any source.

### 2.2. Computational science

***Multimodal machine learning:*** In computational sciences, multimodal learning is a well-established research area whereby meaningful information is extracted from two or more modalities and combined to achieve a specific task. The aim is to reduce the semantic gap between more directly accessible features and semantic interpretations (Smeulders et al., 2000) so that the derived semantics can serve as an effective bridge between contributions made in quite different presentation modalities, such as text and image. Bridging the semantic gap is expected to lead to better performance (often involving prediction) in tasks using multiple modalities instead of just one, e.g., sentiment prediction (Poria et al., 2017) from videos using sequences of image frames and audio. With the recent groundbreaking advances in machine learning and the availability of large multimodal datasets, representation learning models can be trained automatically (Ngiam et al., 2011; Chen et al., 2020; Jia et al., 2021; Radford et al., 2021), and their meaningfulness assessed via standard prediction testing benchmarks. A large body of work exists (Baltrusaitis et al., 2019) targeting areas spanning learning algorithms, the fusion of multiple modalities, evaluation metrics and applications that go beyond engineering to domains like medicine and arts. Some noticeable areas and applications with image and text as modalities that have made significant progress in the last decade are image captioning (Kiros et al., 2014; Karpathy and Fei-Fei, 2015; Hossain et al., 2019), cross-modal retrieval (Socher et al., 2014; Xu et al., 2015; Zhen et al., 2019), and text to image generation (Mansimov et al., 2016; Qiao et al., 2019; Ramesh et al., 2021).

***Beyond descriptive and literal relations:*** In the case of most vision and language model learning and benchmark tasks, the surrounding text is always semantically related at a descriptive and literal level to the image. However, this assumption hardly holds for examples commonly found in online news or advertisements. In such media, the presentation modalities can be weakly linked or abstractly related to one another so as to emotionally engage and influence the reader. Social media offer another example where posts are mostly multimodal, with users uploading billions of photographs^1^ with opinions every day on sites such as Facebook and Twitter. Consequently, some researchers (Chen et al., 2013) have investigated Twitter data to distinguish between visually relevant and visually-irrelevant tweets and have built models to predict the two categories. Taking this one step further, Vempala and Preotiuc-Pietro (2019) take inspiration from Marsh and White (2003) and have studied image-text tweets for modeling relations that capture which modality adds information (or not) to the other. There is also work on estimating the semantic correlation (Zhang et al., 2008; Xue et al., 2015) and concept level relatedness between modalities (Yanai and Barnard, 2005) (e.g., between local image regions and words), but researchers have only recently (Chinnappa et al., 2019) started combining inter-modal relationships with computational modeling to investigate relations at a deeper level.

Zhang et al. (2018) focus on investigating non-literal relations between visual and textual persuasion for the automatic analysis of advertisements. They divide the relations into *parallel* vs. *non-parallel*, e.g., when the image and text convey the same message independently (but not necessarily exhibiting literal overlap) and when the meaning is ambiguous or incoherent if the image and text are combined, respectively. However, one difficulty with communication science taxonomies is that their level of detail sometimes makes it difficult to assign a particular class to an image-text pair, especially for an untrained analyst. Recently, to address such issues, both Kruk et al. (2019) and Otto et al. (2019b) have extensively combined image-text taxonomies from semiotics with metrics from computational science and proposed interpretable and computable categories for image-text relations. Inspired from semiotics, Kruk et al. (2019) focused on investigating Instagram posts from three perspectives: author intent, contextual or literal meaning overlap, and signified meanings (meaning multiplication) between the image and text. Their contextual and signified relations are inspired by the semiotically-inflected proposals of Kloepfer (1976) and Marsh and White (2003), respectively. The authors report noticeable gains in multimodal intent classification when the interpretation of image and text diverges. Alikhani et al. (2020) introduce a new dataset and study different types of cross-modal coherence relations (such as subjective, story, meta) between image-text pairs. Most recently, Utescher and Zarrieß (2021) studied types of referential relations between image and text in long text documents such as Wikipedia articles and posed a new research direction for vision and language modeling frameworks. Similarly, Sosea et al. (2021) show the use case of modeling image-text relationships (*unrelated, similar, complementary*) to improve multimodal disaster tweet classification.

***Image-text computational metrics:*** Whereas the approaches above generally attempt to provide classification in terms of text-image relations directly, it has also been proposed that more widely applicable and accurate characterizations might be gained by drawing on more robust metrics derived from the text, image, and their inter-relations from which more specific text-image relations can be derived. Henning and Ewerth (2018), for example, propose two metrics to characterize image-text relations: *cross-modal mutual information* (CMI) and *semantic correlation* (SC). For a given image-text pair, CMI (with values ranging between 0 and 1) measures the number of shared real-world objects, entities and concepts, while SC (ranging between -1 and 1) measures how much meaning is shared between the two modalities. In contrast to the common assumptions (Parekh et al., 2021) made in several works including image captioning and image-text synthesis where image and text are always semantically related, the semantic correlation here can also be negative, which means relations where the overall meaning is incoherent can also be captured. Similarly, Otto et al. (2019a) propose a further metric called the *abstractness level* (ABS), which measures whether the image is an abstraction of the text or vice versa.

Otto et al. (2019b) also propose a taxonomy of eight image-text relations derived from Barthes (1977) and Martinec and Salway (2005), mapping the three metrics SC, CMI and STATUS to distinctive image-text classes. While CMI and SC measure the information and meaning overlap, STATUS, as introduced in Section 2.1.1, measures the relative importance of an image and text. In addition to the three positively correlated categories termed as *complementary, illustration* and *anchorage*, there are three negatively correlated (*contrasting, bad illustration, bad anchorage*), and two image-text relations with no information overlap (*interdependent, uncorrelated*), which may be deployed in domains such as advertisements and social media to catch the user's attention.

***Computational news values and analysis:*** Recent efforts toward estimating and extracting news values have focused almost exclusively on the text. Potts et al. (2015) investigates linguistic techniques such as tagged lemma frequencies, collocation, part-of-speech tags, and semantic tagging to extract news values. The study used a 36-million-word corpus of news reporting on Hurricane Katrina to explore the usefulness of computer-based methods. Bednarek et al. (2021) conduct a similar empirical study of news articles concerning the Australian national holiday “Australia Day” in the Australian press. In addition, di Buono et al. (2017) and Piotrkowicz et al. (2017) focused on developing fully automatic methods for extracting and classifying news values from headline text. di Buono et al. (2017) relied on news value specific statistical features and Piotrkowicz et al. (2017) additionally experimented with word embeddings and emotion labels to train machine learning algorithms such as Support Vector Machines (SVN: Cortes and Vapnik, 1995) and Convolutional Neural Networks (CNN: LeCun et al., 1998). Similarly, Belyaeva et al. (2018) address the problem of estimating news values like *frequency, threshold*, and *proximity* by applying various text mining methods.

For news media research in general, Motta et al. (2020) describe a framework of newsworthy aspects (called news angles) and a data schema to bridge the gap between formal journalism literature and computational capabilities (named entity recognition, temporal reasoning, opinion mining, and statistical analysis). News angles, in addition to signifying the newsworthiness of an event, also provide a predefined theme/structure to report the event. Similarly, some very recent works address the use of technology toward supporting journalistic practices using semi-automated news discovery tools (Diakopoulos et al., 2021), discuss responsible media technology use for personalized media experiences and news recommendation with automatic media content creation (Trattner et al., 2021), and propose the development of AI-augmented software to support citizen involvement in local journalism (Tessem et al., 2022). All these works position the use of recent breakthroughs in image recognition, natural language processing and other AI technologies for automated or semi-automated news discovery, content creation, and improving user interaction and accessibility. In addition, problems of bias in automated systems, fake news, privacy, news literacy and societal challenges are also discussed and considered important challenges in the ever-changing landscape of technology and information accessibility. However, the role of news angles and news in general in the context of modalities other than text has yet to be explored or discussed.

## 3. Proposed framework for multimodal news analysis

In this section, we introduce and discuss our framework for multimodal news analysis drawing motivation from the related work. We bring together different analytical perspectives that have either been used (such as news values) or not yet explored (image-text relations) to analyse news. From computer science perspective, most tasks in multimodal machine learning with respect to news revolve around fake news detection (Giachanou et al., 2020; Singh et al., 2021), news image captioning (Liu et al., 2021), geo-location estimation (Zhou and Luo, 2012; Tahmasebzadeh et al., 2022), and source or popularity prediction (Ramisa, 2017).

**Figure 2:**
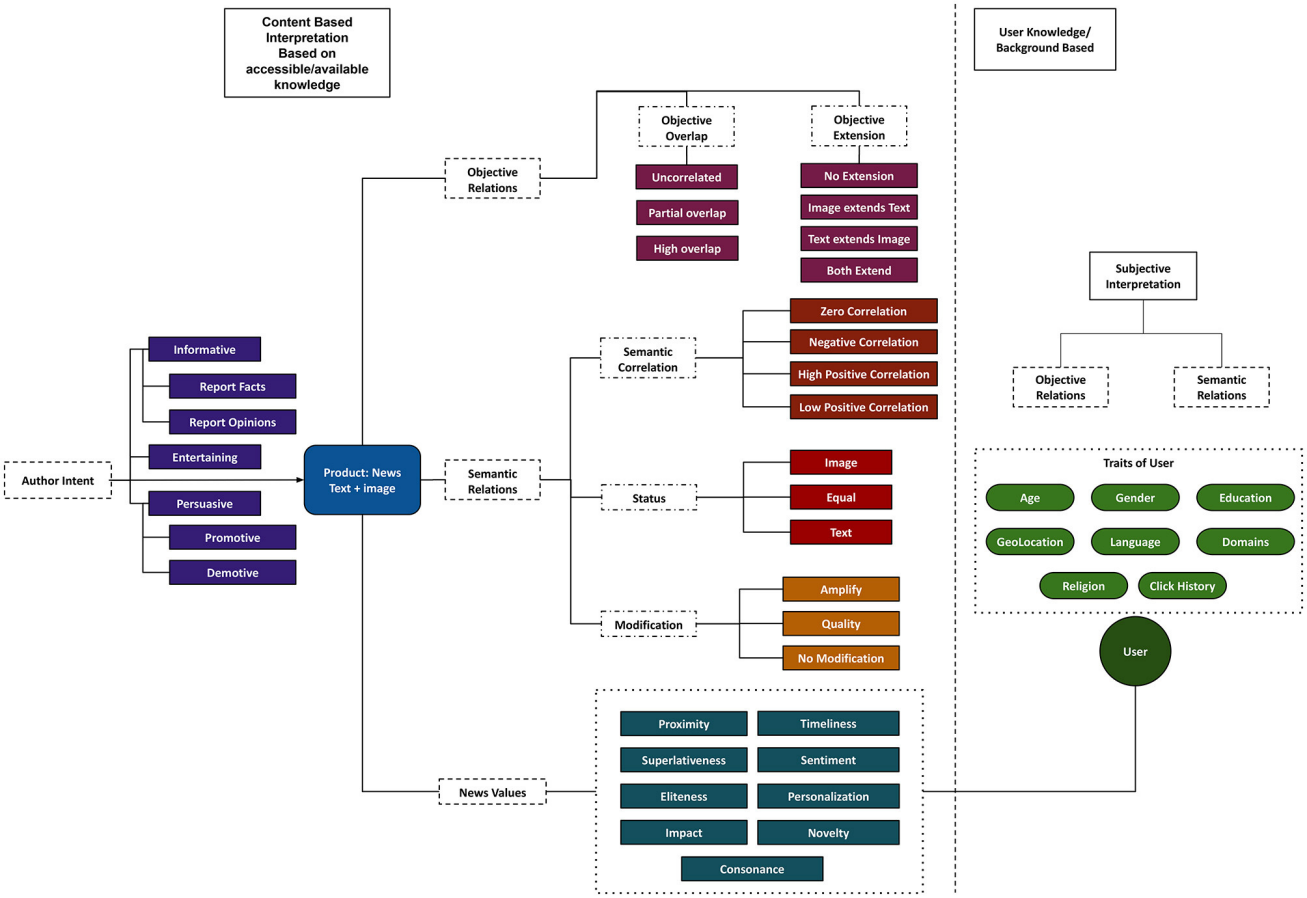
Proposed framework with different perspectives and classes for multimodal news article analysis.

Figure 2 offers an overview of our news analysis framework, covering:

(a) The news production aspects of author intent and news values signifying communicative purpose and newsworthy elements in the news article, respectively,
(b) Multimodality aspects of different image-text relations that signify the interplay of image and text and their use in news, and
(c) The news consumption aspect of subjective interpretation, which explains how user modeling and the interpretation connect to the other two aspects given in (a) and (b).

With our framework, we intend to enable new tasks on large-scale data that can aid in discourse analysis and be of interest to researchers in computational science, communication and media studies. Recent work on news values (Caple, 2013; Caple et al., 2020) has progressed from text-only to image and text both, which ultimately begs the question of how these modalities need to be related to one another (image-text relations). However, this aspect has not yet been considered in combination with news values. Therefore, a framework that can provide a view of all these aspects is going to be far more revealing in discourse analysis than any approach restricted to just one aspect. In the next sections, this is demonstrated by the discussion of several examples and case studies.

Next, we first summarize the individual parts of the framework and then explain them in detail with illustrative examples. In this section, we dig deeper into the individual aspects of (a) and (b) while providing detailed examples, and briefly touch upon the interplay between these different aspects and the news consumption aspect of subjective interpretation. The four main areas of the framework are the following:

- **Author intent** captures the author's primary purpose (communicative goal) behind writing the article. A news article has some facts and information, may have other people's opinions, and the author's own opinion and analysis on an issue. For example, the authors' intent could be just to inform readers about an event or persuade them by making them align with an idea in the article. In the field of rhetorical criticism and genre analysis, intent corresponds to the communicative purpose of a text (Swales, 1990; Miller, 1994). The intent classes in our framework are drawn specifically from recent work on intent taxonomy in Instagram posts (Kruk et al., 2019); broader classification systems are discussed, for example, by Martin and Rose (2008).
- **Cross-modal relations** capture the interplay between image and text, capturing their use in news articles. In other words, this primarily indicates how image and text are tied together for the story in an article. Various image-text relation taxonomies from semiotics were reviewed above. However, even though these often have detailed definitions and divisions of classes, their categories (Marsh and White, 2003; Martinec and Salway, 2005; Unsworth, 2007) sometimes overlap and are hard to interpret. Conversely, categories proposed in computational science are often distinctive and informative, but are either proposed for a particular domain [like Instagram posts from Kruk et al. (2019)] or mainly to capture the role of modalities (Zhang et al., 2018; Vempala and Preotiuc-Pietro, 2019) and generalized image-text relationships (Otto et al., 2019b). In our framework, we both re-purpose existing relations with clearer definitions of classes and scope and introduce new relations specifically for news.
- **News values** capture the news-centric attributes defined in the journalism literature that we introduced in Section 2.1.2. As explained earlier, news values provide crucial insights into how some events are packaged as “news” by news media. We mainly adopt the news values from Caple et al. (2020) and, when needed, extend them with further categorization to make them more inclusive and discrete. In particular, we also review computational approaches that can be used to detect news values from image and text.
- **Subjective interpretation** capture the user-centric changes in the aspects discussed above. As any article is written for a target audience, the demographics and their characteristics certainly have some influence on the use of imagery and language in a news piece. For this reason, we provide an additional dimension of classification that allows us to link cross-modal relations and news values with certain codified aspects of the user's background. We term this the ‘subjective interpretation'. Although we do not delve into this aspect in detail, it allows for the possibility to do comparative studies given analytical aspects above and user characteristics.

In the next sections, we elaborate on each of these aspects in the same order as introduced above, propose additions and modifications, explain the difference to existing literature, and provide real-world examples to motivate the use of the proposed categories.

### 3.1. Author intent

As briefly described before, *Author Intent* represents the author's main purpose behind the news article. Recently, Kruk et al. (2019) propose an intent taxonomy for Instagram posts centered around the presentation of self (Hogan, 2010; Mahoney et al., 2016). They propose eight categories, which are: *advocative, promotive, exhibitionist, expressive, informative, entertainment, provocative:discrimination*, and *provocative:controversial*. While independent journalists' and authors' intent can be placed under some of these categories, we are more interested in intent arising as a combination of author, the idea of the article and the publisher. Thus, the categories that strictly represent presentation of self (*exhibitionist, expressive*) are in general less significant intents for news. Categories under provocation, on the other hand, are related to hate-speech and propaganda, which is certainly an interesting area on its own right in computational analytics.

Building on this, we propose a more structured two-level taxonomy for capturing author intent. *Informative, Entertaining* and *Persuasive* are the top-level categories. *Informative* means that the author's main purpose is to inform the reader about news via unbiased reporting of facts and information. The articles under this category are descriptive and expository focused on details, facts, eye-witness accounts and opinions/arguments of others (such as public figures or people in general) and linguistically distinguishable as aligning along some of the distinct dimensions of registerial variation described in detail by Biber (1988). *Entertaining* articles, in contrast, refer to satirical or spoof pieces that often take a humorous jab at organizations or people that hold power. Finally, *Persuasive* refers to an author supporting or refuting a policy, person, organization or broadly an idea in the article *via* personal opinions, interpretations, point of view or judgements. Not all the intent categories are mutually exclusive, and so an article can have more than one author intent. These diverse properties naturally suggest that corresponding computational language models differentiate among the text types and registers represented.

**Figure 3:**
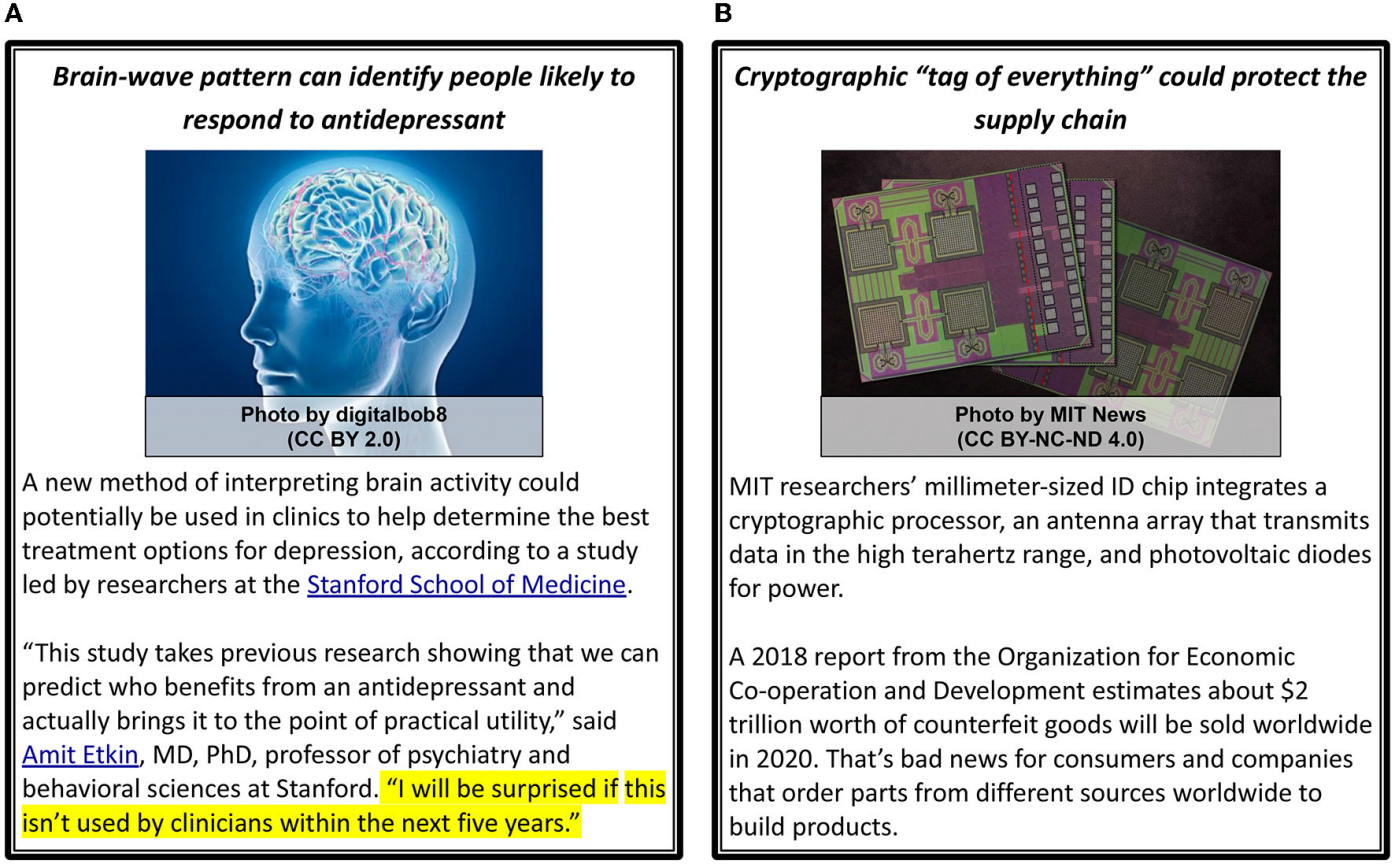
*Author Intent* examples for *Report Facts* and *Opinions*. Left: the portions of the text (quotes) that lead to the assignment of *Opinions* are highlighted (yellow). Both articles include factual information, and thus the category *Facts* is assigned. **(A)** Facts and Opinions. **(B)** Facts.

Developing the account further, *Informative* is then divided into two categories:

- *Report facts*: When the article mainly focuses on what (description of the event/news), when (time, date) and where (location) of the news/event. For example, a news article about a hurricane with the description of wind speed, amount of rain, time and duration, location of landfall and the details of destruction and damage it caused. A real news factual example is shown in Figure 3B, where the article is about a new chip, its components and the benefits. The articles under this category can also be an introduction of a problem and possible solutions to the readers. For example, a news about water contamination in the region and the solutions recommended by health authorities.
- *Report opinions*: In this case, articles contain references to other people's opinions, arguments and comments, which sometimes in text refers to quoted snippets. For example, a news article with references to people questioning the government about their ill-preparedness for the hurricane. In Figure 3A, another news example can be seen which is scientific and factual, but also consists of comments and opinions of another person, e.g., a professor, given in quotes. The examples show that not all quoted snippets are opinions, as the first quoted snippet is a summary of the research rather than an opinion.

**Figure 4:**
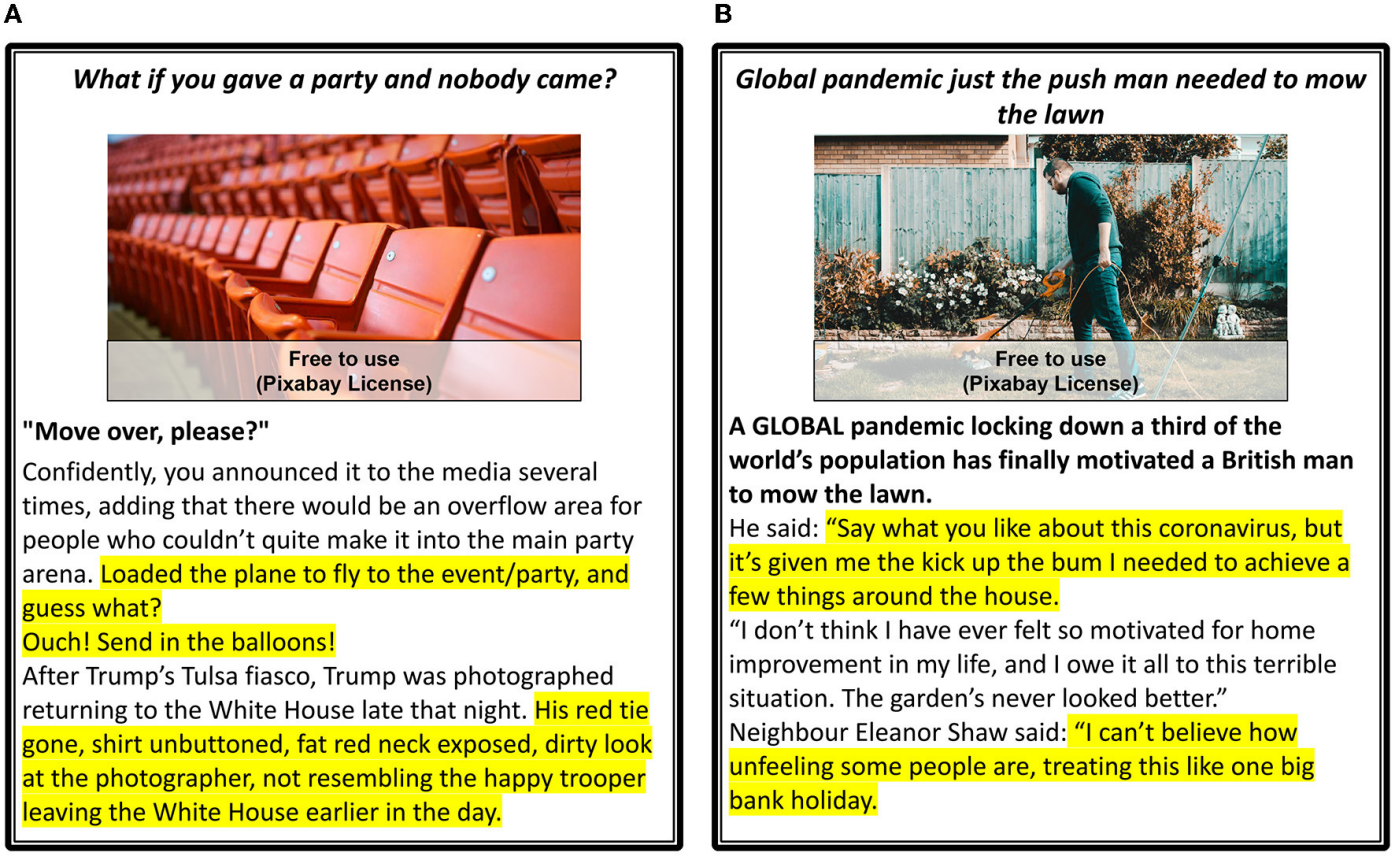
*Author Intent* examples: Entertaining and Opinions. The portions of the text (entertaining, satirical parts, or quotes) that lead to the assignment of corresponding categories are highlighted (yellow). **(A)** Entertaining. **(B)** Entertaining & Opinions.

*Entertaining* news articles rely heavily on irony and humor, and it is almost impossible to mistake them as serious news. This is different from fake news, which intentionally misleads people into believing that the story is real (Golbeck et al., 2018). The articles here mimic real news but still cue the reader that they should not be taken seriously. Examples of this category are news articles from satire websites like *thespoof.com, thecivilian.co.nz* and *thedailymash.co.uk*. Two examples from such websites are shown in Figure 4, where highlighted text shows informal language that is not used in actual news articles. Interestingly, the images used in these examples are neutral and do not reflect the entertaining intent of the article.

**Figure 5:**
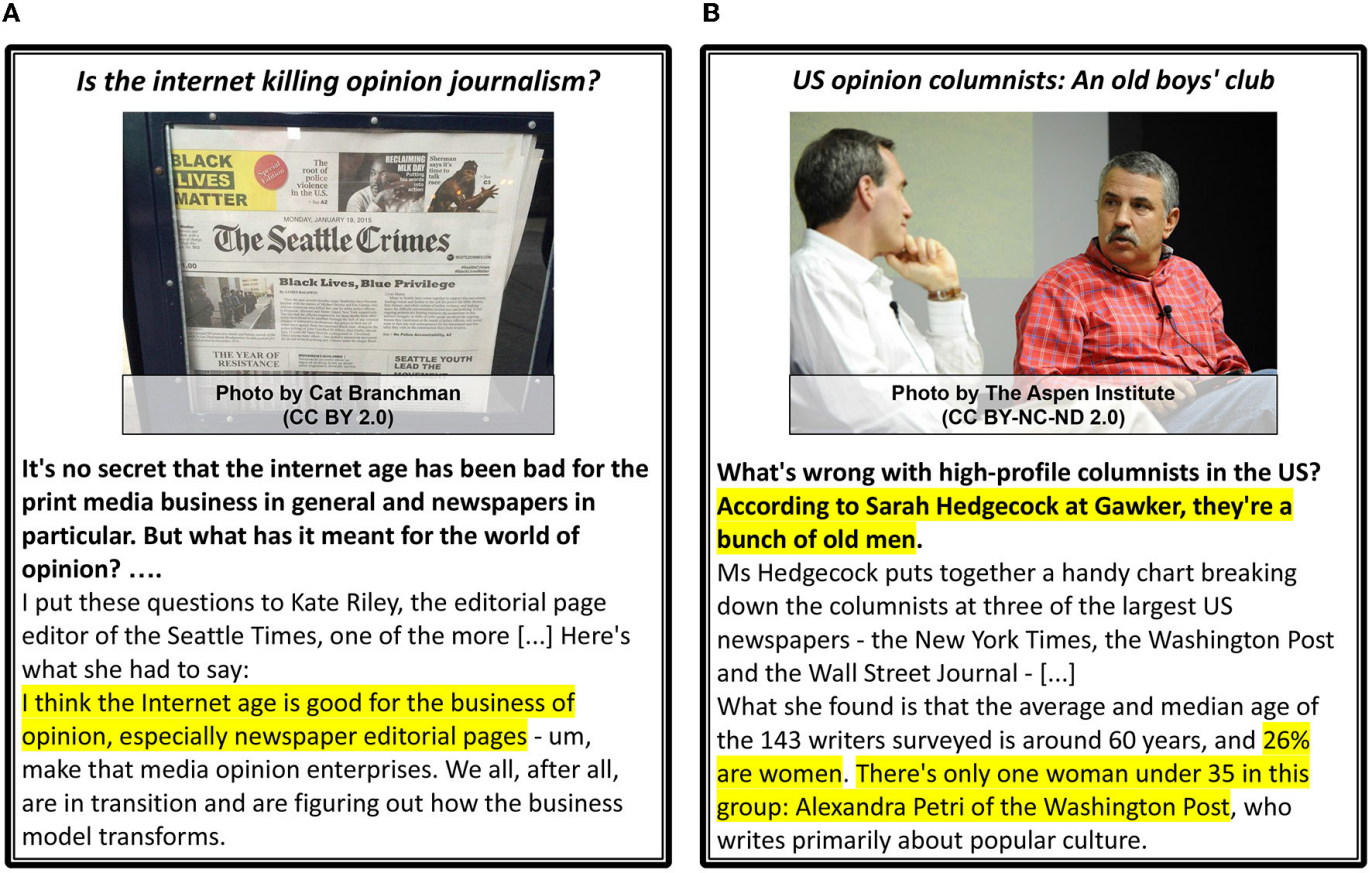
*Author Intent* examples for Persuasive–Promotive and Persuasive–Demotive. The portions of the text that lead to the assignment of corresponding categories are highlighted (yellow). **(A)** Promote. **(B)** Demote.

*Persuasive* articles are written with the purpose to persuade and convince the readers to consider or accept author's opinion, position and point of view. Opinion pieces and articles fall under this category. It is also a possibility that an article holds no position and so only falls under the *informative* category. If not, *persuasive* is divided into two opposing sub-categories: *Promote* and *Demote*, defined with respect to the core theme or idea of the article. *Promote* refers to when an author aligns with or supports a policy, person or organization and, in so doing, aims to persuade the reader to do the same. *Demote* is the converse of this. We show two examples of such news articles in Figure 5, where article (a) is optimistic about the Internet for opinion journalism, while article (b) is about press culture in the US and the lack of gender diversity. Additionally, use of harsh language or tonality, attacks toward someone, presence of controversy and propaganda are all possibilities within such articles. These establish important targets for further research but are not yet explicitly categorized under our taxonomy.

### 3.2. Cross-modal relations

Our proposed taxonomy of cross-modal relations (last column) is inspired from multiple taxonomies. Table 1 shows a comparison of taxonomies proposed in the computational science literature that draw on semiotics. The top three rows in the table represent relations where the image and text are coherent and belong together, and relations in the next three rows are where the image and text differ in meaning and are incoherent. While the top three relations can be represented in both Kruk et al. (2019) and Vempala and Preotiuc-Pietro (2019), the differences between relations based on modality's importance cannot be established uniquely. Moreover, Vempala and Preotiuc-Pietro (2019) cannot represent any of the relations where either image and text are incoherent or related on an abstract level (row 7 in Table 1) without sharing any information. Whereas, Kruk et al. (2019) can represent the incoherent relations, all ambiguously under one combination of its contextual and semiotic classes. As our relation taxonomy is inspired from Otto et al. (2019b), all the relations in the leftmost column can be uniquely represented by our taxonomy (rightmost column). In addition, we have extensions of CMI and SC to explicitly model the presence of additional information and fine-grained relations, respectively.

**Table 1:** A comparison of taxonomies proposed in computational sciences to build multimodal models.

**Otto et al. (** **2019b** **)**		**Vempala and Preotiuc-Pietro (2019)**	**Kruk et al. (** **2019** **)**		**Our taxonomy**	
Anchorage		Text is represented Image adds	Contextual : Semiotic : Transcendent Parallel/Additive	OO	Partial - High
CMI	> 0			OE	Image extends text / Both
SC	> 0			SC	High-positive
STATUS	Image			STATUS	Image
Illustration		Text is represented Image does not add		OO	Partial - High
CMI	> 0			OE	Text extends image / Both
SC	> 0			SC	High-positive
STATUS	Text			STATUS	Text
Complementary			Contextual : Semiotic : Close Parallel	OO	High
CMI	> 0			OE	Both extend
SC	> 0			SC	High-positive
STATUS	Equal			STATUS	Equal
Bad Anchorage		Not represented	Contextual : Semiotic : Minimal Divergent	OO	Partial - High
CMI	> 0			OE	Image extends text/both
SC	< 0			SC	Low-positive/negative
STATUS	Image			STATUS	Image
Bad Illustration				OO	Partial–high
CMI	> 0			OE	Text extends image/both
SC	< 0			SC	Low-positive/negative
STATUS	Text			STATUS	Text
Contrasting				OO	High
CMI	> 0			OE	Both extend
SC	< 0			SC	Low-positive/negative
STATUS	Equal			STATUS	Equal
Interdependent			Contextual : Semiotic : Minimal Additive	OO	No Overlap
CMI	= 0			OE	No extension
SC	> 0			SC	High-positive
STATUS	Equal			STATUS	Equal
Uncorrelated		Text is not represented Image does not add	Contextual : Semiotic : Minimal Divergent	OO	No Overlap
CMI	= 0			OE	No extension
SC	= 0			SC	Zero correlation
STATUS	Equal			STATUS	Equal

OO, Objective Overlap, OE, Objective Extension, CMI, Cross-modal Mutual Information, SC, Semantic Correlation.

We suggest additions and modifications for object- and semantic-level relations along with news-centric attributes for richer news analysis. We define these relations building on measures established in previous work. However, while relations in other taxonomies can be broken down (sometimes not distinctively) at conceptual, semantic and relevance levels, our framework is more fine-grained. Furthermore, our framework can be applied to any image-text pair, while at the same time offering relevant relations and attributes specifically for news analysis.

There are then two types of high-level cross-modal relations in the framework, Objective relations and Semantic relations, which are explained in detail in following two Sections 3.2.1 and 3.2.2.

#### 3.2.1. Objective relations

**Objective relations** measure the amount of shared real-world entities or concepts between the two modalities (image and text). Information for objective relations refers to real-world objects and named entities, such as persons, locations and landmarks, actions, events, scenery, organizations, products. We use the idea of imageability (Kastner et al., 2020) here to differentiate between abstract and concrete entities/concepts. Under objective relations, entities or concepts have either concrete iconic depictions or give a clear mental image. In addition, information can also refer to concepts like scenes and actions, which have a concrete mental image. Rest of the named entities and concepts which have no concrete depiction are considered in semantic relations. For example, the term religion can have various possible visuals (symbol, church/temple), but is still an abstract concept. In contrast, a specific religious ceremony like baptism has a far clearer – i.e., more reliably associated – mental image and concrete depiction. This is specifically true for certain named entities in text such as large numbers, season and time, for which there is no clear depiction. Previous works mix concrete and abstract concepts under one relation, such as “text is represented” relation in Vempala and Preotiuc-Pietro (2019), “contextual” relation in Kruk et al. (2019), and Otto et al. (2019b) referring to entities as “main objects” in foreground of an image. From the computational perspective, object (Deng et al., 2009; Thomee et al., 2016), scene (Xiao et al., 2010; Zhou et al., 2018), event (Xiong et al., 2015; Müller-Budack et al., 2021a), and action recognition (Heilbron et al., 2015; Gu et al., 2018) models and their classes provide the list of all concepts and entities that have clear depictions and are thus identifiable in an image.

It is further divided into two classes: *Objective Overlap* that signifies mutual information between two modalities (similar to CMI), and *Objective Extension*, a new aspect, that indicates the modality contributing more entities and concepts.

**Figure 6:**
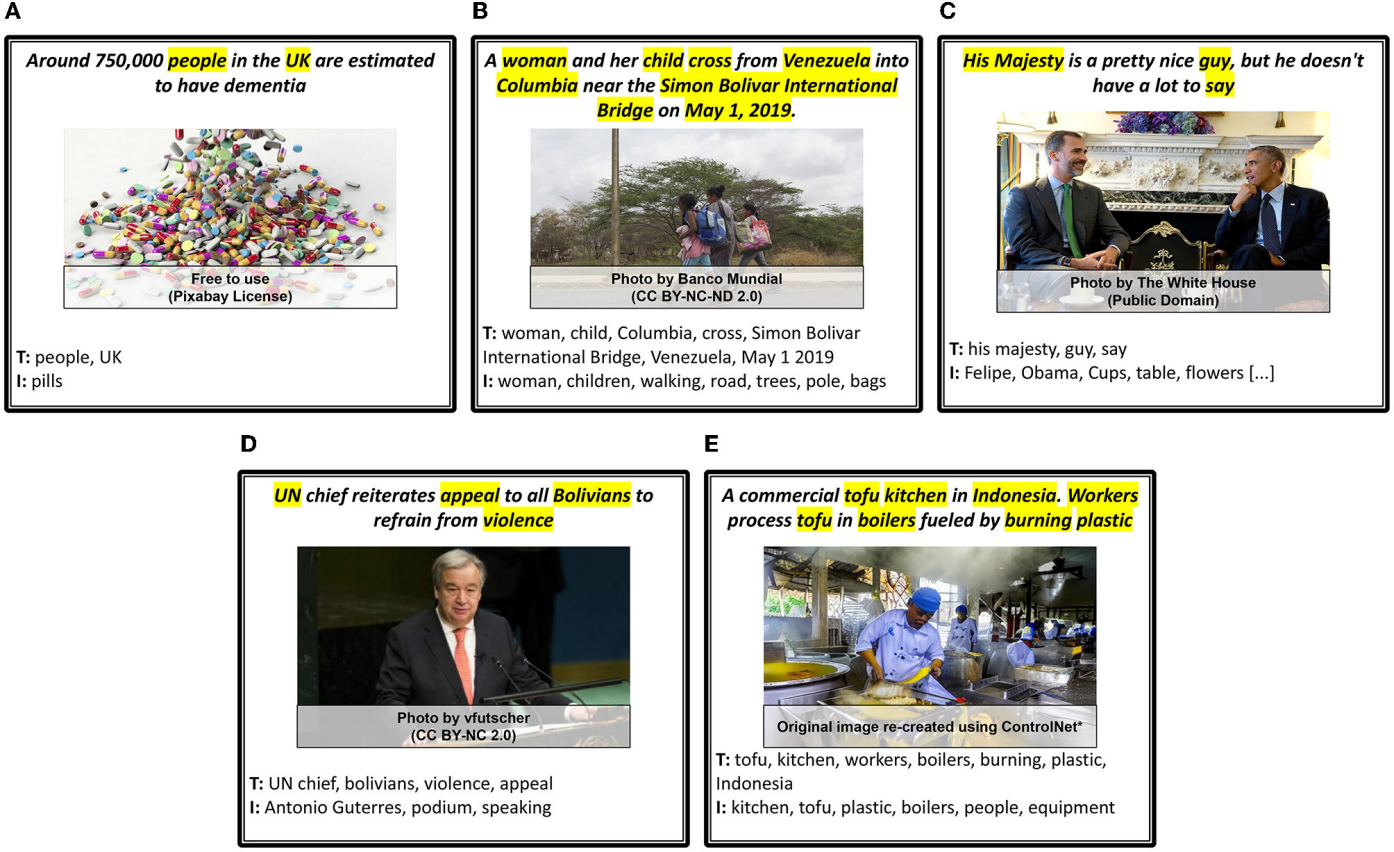
News articles annotated with the sub-categories of *Objective relations*. Each sample includes the identified textual (**T**) and visual (**I**) concepts, entities, etc., along with the assigned sub-categories of *Objective Overlap (OO)* and *Objective Extension (OE)*. *Used ControlNet (Zhang and Agrawala, 2023) to re-create images because of licensing issues. **(A)** OO: Uncorrelated, OE: No Extension. **(B)** OO: Partial Ovelap, OE: Both Extend. **(C)** OO: High Overlap, OE: Image Extends Text. **(D)** OO: Partial Overlap, OE: Text Extends Image. **(E)** OO: High Overlap, OE: Both Extend.

In practice, to model these relations and to identify the correct sub-categories, we need some specific ranges of values: (i) the set of entities or concepts in an image (*N*_*I*_), (ii) the set of entities or concepts in the text (*N*_*T*_), and, (iii) the set of entities or concepts that overlap between image and text (*N*_*O*_). Examples of image-text pairs from news articles are shown in Figure 6. They include image-headlines and image-captions pairs, respectively, to explain the categories. Both categories of objective relations should also be beneficial and informative in the case of informal news sources such as Twitter, where text has a character limit resulting in much information being packed into a few sentences.

***Objective overlap (OO)*** measures the overlap between a multimodal news article's image and text components. The purpose is to establish material and conceptual links between one or multiple images and text in a news article. It measures the intersection of information from two presentation modalities (image and text) and can be an actual number or a quantitative value signifying the extent of overlap (as in CMI). In our framework, this is divided into three further sub-categories:

- *Uncorrelated* stands for cases where none of the defined material or conceptual ties co-exist in the textual and visual content (as when CMI = 0). According to the introduced notation, this is when *N*_*O*_ = ∅. Example (a) in Figure 6 shows an uncorrelated pair, where pills in the image have no shared word or phrase in the text.
- *Partial overlap* is when there is an overlap between the defined concepts in image and text, but not every aspect exists in both modalities (similar to CMI ~0.5), i.e., *N*_*O*_ = (*N*_*I*_∩*N*_*T*_) and 0 < |*N*_*O*_| < *min*(|*N*_*I*_|, |*N*_*T*_|). Examples (b) and (d) in Figure 6 show this overlap, where in both modalities additional concepts are present besides the overlapping concepts.
- *High overlap* is when all the entities or concepts from either modality are present in *N*_*O*_. With our notation, it can be computed as *N*_*O*_ = (*N*_*I*_∩*I*_*T*_) = *min*(|*N*_*I*_|, |*N*_*T*_|)), where *N*_*O*_ = *N*_*I*_ when high overlap is because of image's contribution and *N*_*O*_ = *N*_*T*_ when high overlap is because of text's contribution. Example (c) shows high overlap with all aspects (majesty, guy, say) in text seen in the image. In the third case, when *N*_*O*_ = *N*_*I*_ = *N*_*T*_, there is close to a one-to-one correspondence between concepts in image and text (CMI ~1). A prominent example of this is given by image captioning datasets, where the text exclusively focuses on image content. Figure 6E shows the high overlap between concepts and words like kitchen, tofu, plastic, boilers and workers. Although an high overlap is unlikely when comparing an news text article and an image, there is a possibility of high overlap between the image-caption (or parts of the text) and image-headline pairs – when, for instance, an image acts as strong evidence (reference) for the text, e.g., news about a crime scene or a natural disaster.

***Objective extension (OE)*** indicates which modality extends another based on the amount of information contributed by each modality. The relation is inspired from *Extension* described by Martinec and Salway (2005), where the additional information is new but related information (such as date, country, person's name). Extension is only possible when partial or high objective overlap exists between two modalities. The sub-categories for this relation are described below with their corresponding notations:

- *No extension*: This is possible in two scenarios, (a) *OO* is *uncorrelated* (*N*_*O*_ = ∅), see example (a) in Figure 6, and (b) *OO* is *high overlap* with an one-to-one correspondence between shared concepts and no new related concepts. A typical example for this case is image-caption pairs, with the caption describing the objects and scene as depicted in the image.
- *Text extends image* and *image extends text*: Both classes are possible when *OO* is *partial overlap* or *high overlap* and when one of the modalities has more components. That is |*N*_*T*_|>|*N*_*I*_| for *Text Extends image* and |*N*_*I*_|>|*N*_*T*_| for *Image extends text*. The examples (c) and (d) in Figure 6 show these categories. Elaborating further on example (d), text provides details that the appeal is to Bolivians to refrain from violence, while the image depicts the UN chief speaking on a podium.
- *Both extend*: This case is possible in two scenarios, a) *OO* is *partial overlap* and |*N*_*I*_| = |*N*_*T*_|, i.e. that there are still components in both modalities that provide more details than each on its own, and b) *OO* is *high overlap* and there are still additional entities/concepts in both. Example (e) in Figure 6 illustrates the latter case, where the image shows a dimly lit kitchen with partially clothed workers working and the text provides details such as Indonesia and burning.

#### 3.2.2. Semantic relations

**Semantic relations** cover relationships on the meaning level and overall message of the image-text pair that can go beyond descriptive *Objective relations*. The relations under this category refer to how interpretations and meaning can be constructed from a image-text pair by combining information from both modalities. Typically, understanding the meaning of an image-text pair requires knowledge and a user's ability to identify entities or concepts and how to link them. These relations can be inferred based on publicly accessible knowledge sources (e.g., encyclopedia, knowledge graphs, etc.). The exploitation of such sources would be required for computational approaches to identify semantic relations. *Semantic relations* include three different aspects of capturing relationships on the meaning level: *Semantic Correlation* captures whether image and text belong together, *STATUS* signifies modality importance in terms of context and information, and *Modification* indicates whether one modality changes or strengthens certain aspects provided in the other modality. Please note that there might be a semantic relation between image and text, while there is no *objective relation*. For example, Figure 6A shares no entities or concepts, but 750,000 people in text is abstractly linked to large number of pills in the image.

***Semantic correlation*** measures the correlation between image and text. It can refer to concrete entities and contextual, interpretative correlations, that is, how much meaning is shared between modalities according to the SC metric from Otto et al. (2019b). In addition to Otto et al.'s (2019b) SC metric, we replace *Positive correlation* with two sub-classes *Low-positive correlation* and *High-positive correlation* to capture more fine-grained relations. The *Objective relations* only tell us about the objective overlap between image and text, and it remains a possibility that, even with high overlap, the items related can be opposite or contradictory due to context differences at a semantic level. To capture semantic overlap, we include *semantic correlation* in the framework to measure meaning overlap regardless of the shared information.

**Figure 7:**
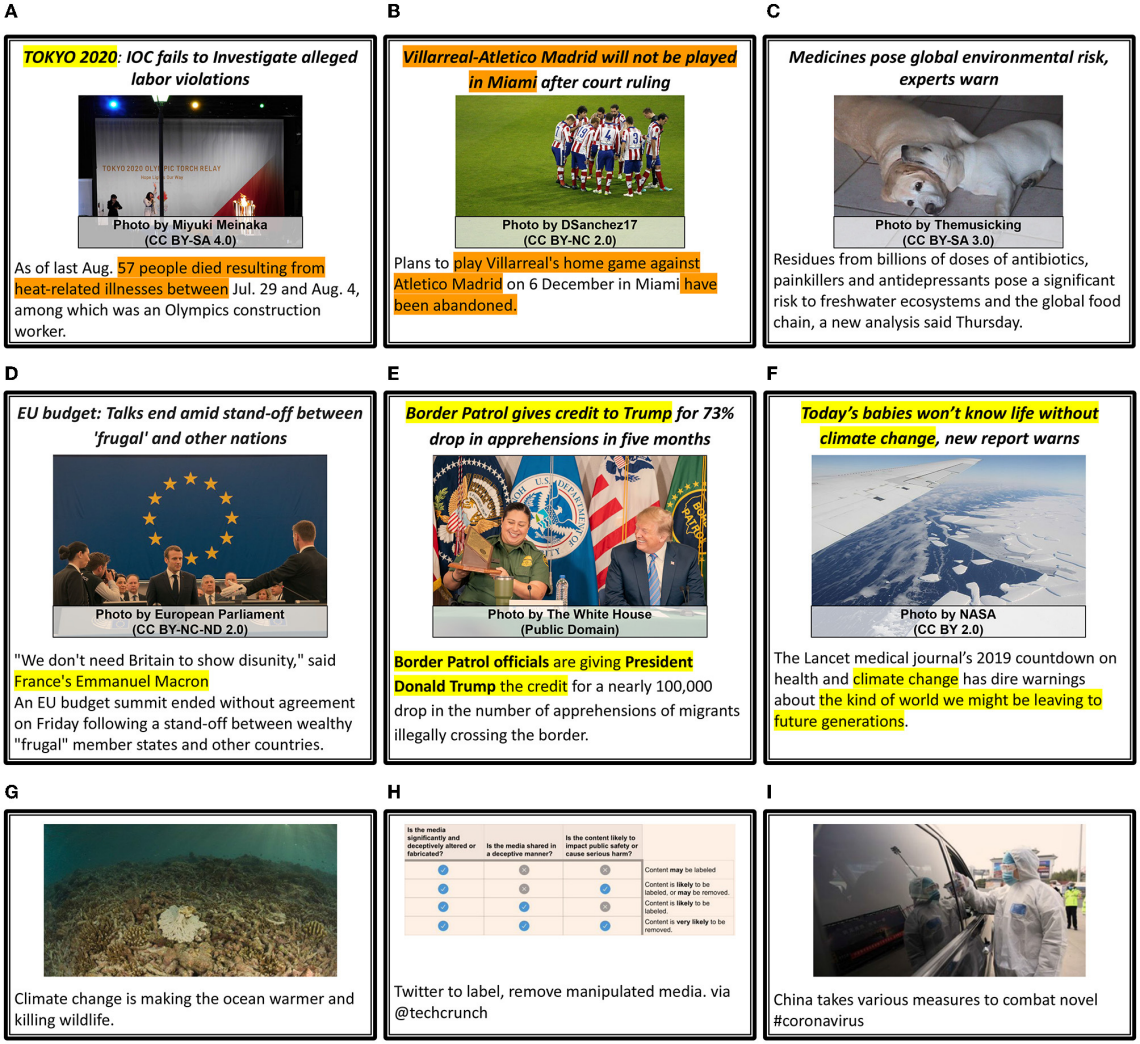
*Semantic Correlation* (SC) and *STATUS* examples. Negative correlation examples have highlighted portion in text (orange) that is opposite in meaning to the image. Low and high-positive correlation examples have highlighted portion in text (yellow) that is aligned in meaning to the image. **(A)** SC: Negative correlation. **(B)** SC: Negative correlation. **(C)** SC: Zero correlation. **(D)** SC: Low-positive correlation. **(E)** SC: High-positive correlation. **(F)** SC: High positive correlation. **(G)** STATUS: Equal. **(H)** STATUS: Image. **(I)** STATUS: Text.

As a result, we divide *semantic correlation* into four sub-categories:

- *Zero correlation*: If the two modalities do not have any material, conceptual, abstract or contextual semantic links, then the image-text pairs are considered as unrelated. We provide a sample in Figure 7C where the given image and the text in the news article do not have any implicit or explicit meaning relations.
- *Negative correlation*: This is the case when information in two modalities has different, opposing or contradictory contexts that disturb the overall meaning of the image-text pair. For example, in Figures 7A, B, two samples of negative semantic correlation are shown where the images contradict the news or information provided in the text. On the left, we have a celebratory image of people with a Tokyo 2020 banner, while the news is about alleged labor violations and resulting deaths. In the middle, we again have an image of football players celebrating that resembles the action of playing the match, whereas the text in news article is about an abandoned match.
- *Low-positive correlation*: In this case, image and text are partially linked by sharing one or a few aspects but not the entire message. Typically, images and text are weakly linked in news articles and have low-positive semantic correlation when images in the news article serve no purpose besides being a placeholder or a stock photograph. For example, in Figure 7D, the image of *Emmanuel Macron* with *EU* countries' flags beside him is weakly linked since the actual news is about *EU budget*.
- *High-positive correlation*: This is the case when image and text belong together through sharing information and the entire contextual message. However, sharing information, i.e., partial or high *objective overlap*, is optional. Particularly topics such as politics, sports and breaking news have images that can be strongly linked to the news text or the headline. Moreover, satire news can have abstract (aesthetic or artistic) images with zero or very low objective overlap but with high semantic overlap with the text. For example, in Figure 7E, the highlighted text describes the people, image scene, and also provides an additional context of the place and event. Similarly, the example in Figure 7F mentions the effects of climate change on future generations while the image shows the melting ice blocks as a result of climate change.

The ***STATUS*** relation indicates the relative importance of image and text with respect to the overall message. Although the text is usually the main modality in a news article [although Stöckl et al. (2020) describe an emerging genre where this is not the case, identifying the relative importance of a modality (image or text)] can be significant to analyse the importance of images against headlines, captions, short text pairs (social media) or the whole news body text. Therefore, we use the *STATUS* relation as defined by Otto et al. (2019a) for modeling image-text relative importance. Relative importance is in terms of meaning and the modality central to the overall message of an image-text pair, in contrast to *extension*, which operates in terms of individual concepts. The *STATUS* relation describes the hierarchical relation between image and text to signify whether they are equally important for the overall message or one modality is subordinate (provides additional information while the main message exists in the other modality) to the other.

*STATUS* has three sub-categories as follows:

- *Equal*: Both parts are equally important for the overall message. There is a possibility that image and text are tightly linked here on the level of *objective overlap* (*High Overlap*) and *semantic correlation* (*High-positive Correlation*). Both can also add information to each other that works together for the overall meaning and explanation. A sample in Figure 7G has an *Equal STATUS* relationship. The text mentions the effects of climate change while the image shows how the coral reefs are dying. The information in each modality is unique and equally important with regard to the overall message of *climate change*.
- *Image*: Here, the image is the center of attention and provides additional information or context not present in the text. The text acts as a caption or explicitly references the image, indicating that the image holds vital information regarding the message. It is still possible that text adds context here and fixes the intended meaning of the image-text pair. The sample in Figure 7H is such an example where the table in the image contains more information since the text describes the overall message without any details.
- *Text*: In this case, the image plays a minor role or is exchangeable with another image. The text provides the main context and other unique and important details relevant to the main message. This is the usual case of news articles, where text holds the main information, and one or more images act as illustrations of a few things in the text. The sample in Figure 7I has the main content in its text, and the image serves as a visualization of the message. The image can be replaced with another one, and the image-text pair's overall message would not change. Thus, the given image-text pair sample has *Text* as *STATUS*.

***Modification*** indicates if one modality changes a particular aspect or meaning in the other. Such aspects can be *sentiment*, emotion, quantities, or broad modifiers (adjectives) present in image or text. Unlike *semantic correlation, modification* is directional in nature because it changes or enhances the meaning or interpretation of the other modality's content. It is relevant for news in particular, where the image's role (like amplifying the news) can be explained *via modification* in addition to news values. The relation is motivated from Bateman's (2014) explanation of Kloepfer's (1976) taxonomy that covers additive image-text relationships. Although Kloepfer defines modification and amplification as two different relations under additive relations, the taxonomy and terminology are still debatable as amplifying any aspect also means changing it (hence modifying it).

**Figure 8:**
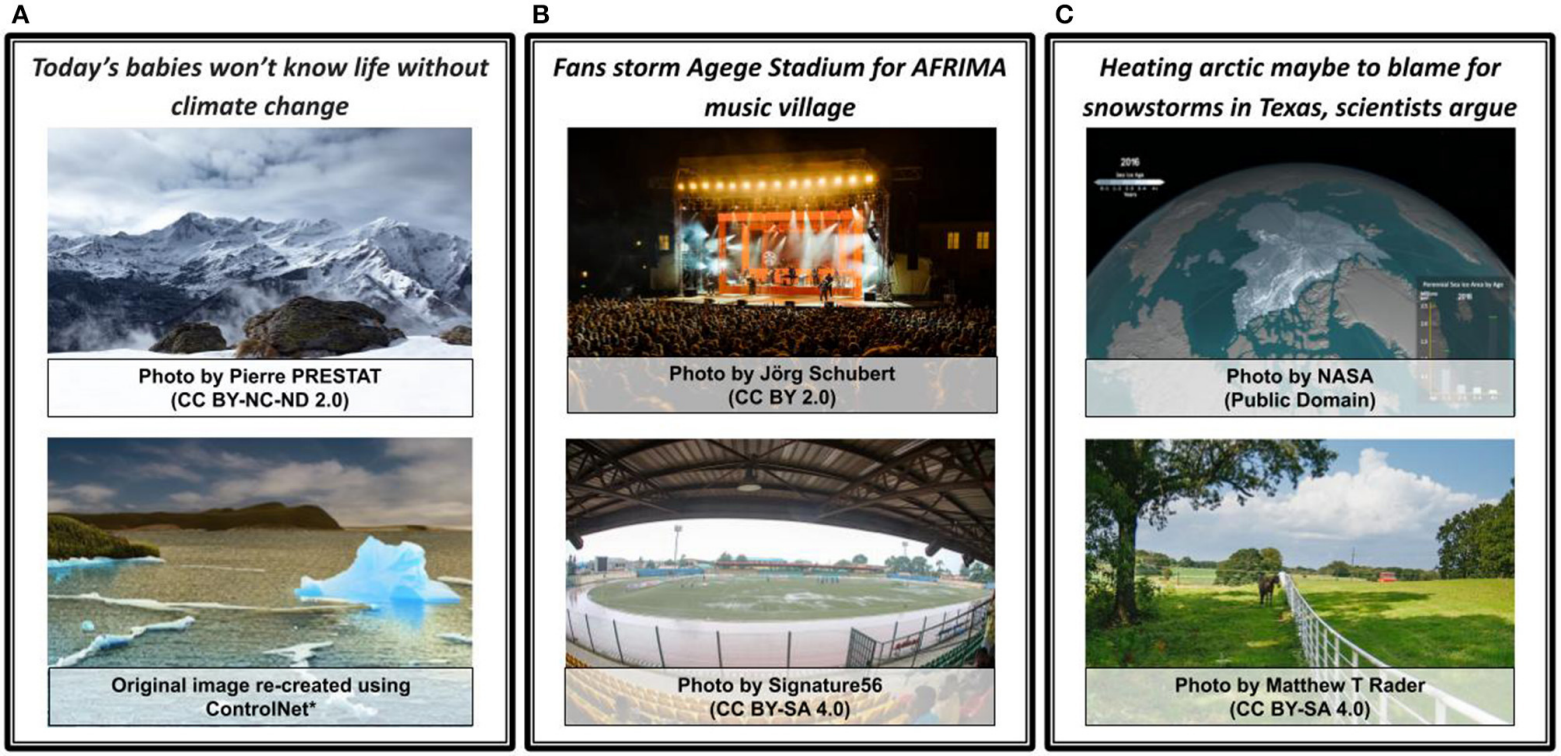
*Modification* examples. Three news headlines each with two different images showing the change in modification relations. *Used ControlNet (Zhang and Agrawala, 2023) to re-create images because of licensing issues.

We define the *modification* relation as a parent relation under which several types of modification can be distinctly sub-categorized as follows:

- *Amplification*: One modality changes the conveyed message by making it, i.e., its meaning or certain aspects in the other modality, stronger (“louder”). Here, the meaning and categories of certain aspects stay the same, but the intensity is increased in the same direction. The bottom image in Figure 8A amplifies the central message in the text, i.e. of exposed landscape due to melting ice contrary to the small iceberg (broken from glacier), which future generations will not get to see and experience. In contrast, the message in the same text is not modified by the top image in Figure 8A since it does not show the drastic changes (drastic melting of snow) as a result of climate change. Similarly, the top image in Figure 8B amplifies the phrase “fans storm” by showing the large number of people gathered for an event mentioned in the text.
- **(A-C)** *Quality modification*: The *Quality modification* changes the conveyed message, its meaning or certain aspects in any way or direction (not the same direction as in Amplify). There is a chance that image and text have a *negative semantic correlation* in such a case. For example, the bottom images in Figures 8B, C are opposite in meaning to “fans storm” and “snowstorms” showing an empty stadium and lush green Texas farms respectively. These examples are specific instances of *Quality modification* due to the stark contrast of focused aspects in the image content when compared to the core theme of the news text.

If neither *Amplification* nor *Quality* modification is present between image and text, then there is no modification. The top images in Figures 8A, C show icy mountains and the arctic, with both images possibly representing melting ice; however, they do not modify the message by either amplifying or changing its quality.

### 3.3. News values

**Figure 9:**
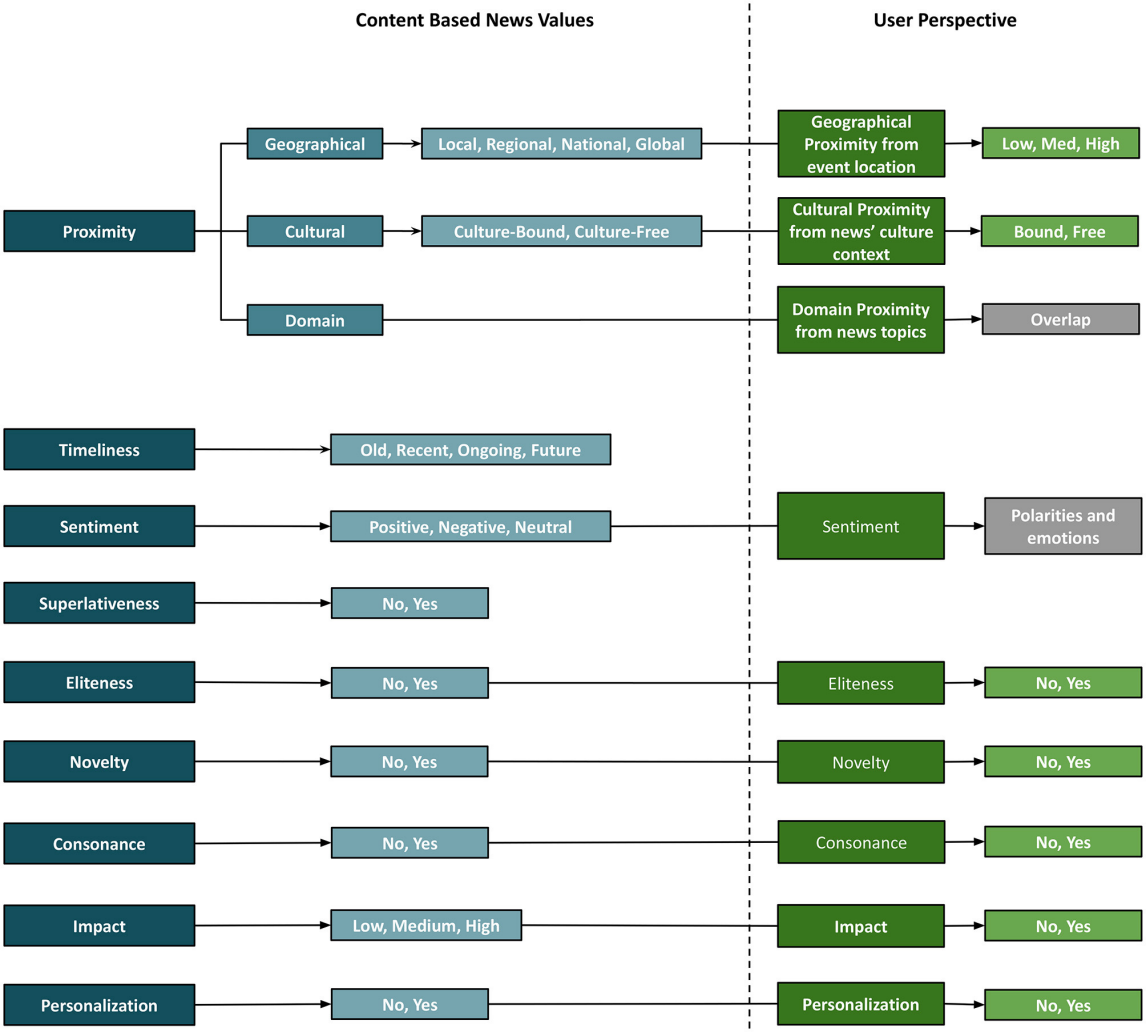
News values from content and user perspective.

In this section, we modify and expand the news values defined by Caple (2013), Caple and Bednarek (2016), and Caple et al. (2020), as they define news values for both image and text in a discursive and content-specific manner, which is interesting and relevant from a multimodal news analytics perspective. We derive new sub-classes for some of the news values to make them more concrete, discrete or meaningful. In our framework, any news value may have the same or different sub-categories for the image and text, which adds another dimension for comparing both modalities. Having said that, where needed both modalities can be used to estimate a single multimodal news value with respect to the news article (explained in each news value). We discuss each news value and its realizations in each modality, define scope and list visual and verbal cues essential to identify them in image and text. In previous work, news values are indicated by their presence or absence, which we also follow for most news values. We leave out the news value *Aesthetics* for two reasons: first, it is only defined for the image, and second, it is a subjective-only and not concretely defined news value. This makes categorizing an image into non-aesthetic vs. aesthetic from only the perspective content a difficult, and probably inappropriate task. In the following sub-sections, we list the news values (see Figure 9) in our framework and their descriptions from the content-only perspective, which can differ from the user-dependent news values covered in *subjective interpretation* in Section 3.4.

**Figure 10:**
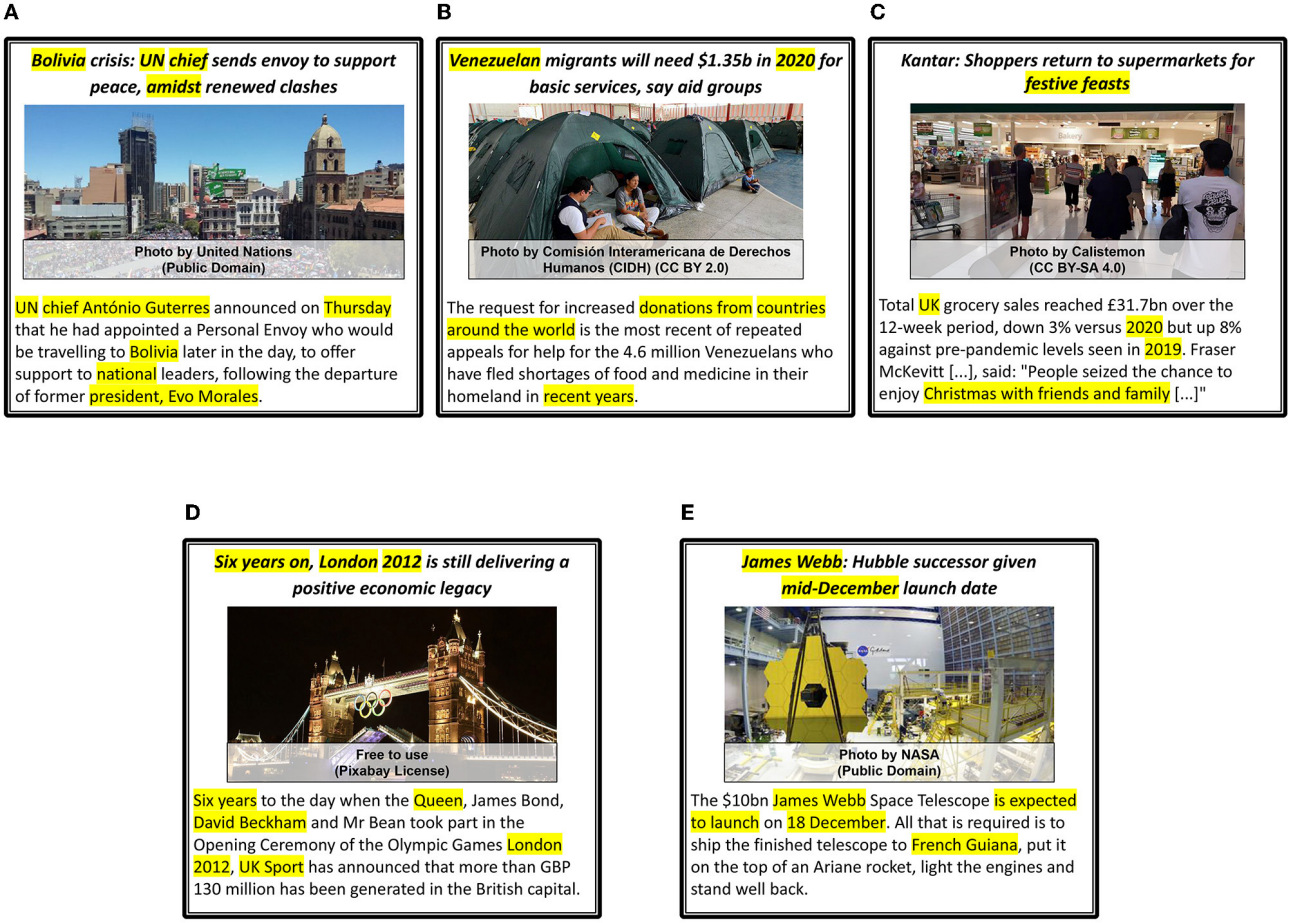
*Proximity, Timeliness*, and *Eliteness* news values examples. All news values except domain *proximity* are described as Category, (textual or visual cues), *[Modalities: I (image), T (Text)]*. The segments in text that mention important words or phrases indicating time, persons, locations, etc. are highlighted (yellow). **(A)** Eliteness : Yes, (UN chief, President), [T], Geo. proximity : National, (Bolivia), [I,T], Cul. proximity : Culture-free, [I, T], Dom. proximity : (world affairs, politics, crisis), [I,T], Timeliness : Ongoing, (Thursday, Amidst), [T]. **(B)** Eliteness : No [I, T], Geo. proximity : International, (Venezuela,World), [T], Cul. proximity : Culture-free, [I,T], Dom. proximity : (migration, refugee-crisis) [T], Timeliness : Ongoing, recent, (2020, recent years) [T]. **(C)** Eliteness : No, [I,T], Geo. proximity : National, (UK) [T], Cul. proximity : Culture-bound, (festive, christmas), [T], Dom. proximity : (shopping, christmas, sales), [I,T], Timeliness : Recent, Ongoing, (2019, 2020), [T]. **(D)** Eliteness - Yes, (Queen, David Beckham), [T], Geo. proximity : National, (London, UK), [I,T], Cul. proximity : Culture-free, [I, T], Dom. proximity : (olympic games, sports), [I,T], Timeliness : Old, (Six years, 2012), [T]. **(E)** Eliteness - Yes, (James webb, NASA), [I,T], Geo. proximity : International, (French Guiana, NASA), [I,T], Cul. proximity : Culture-free, [I, T], Dom. proximity : (space, telescopes, science), [I,T], Timeliness : Future, (expected, 18 December), [T].

**Figure 11:**
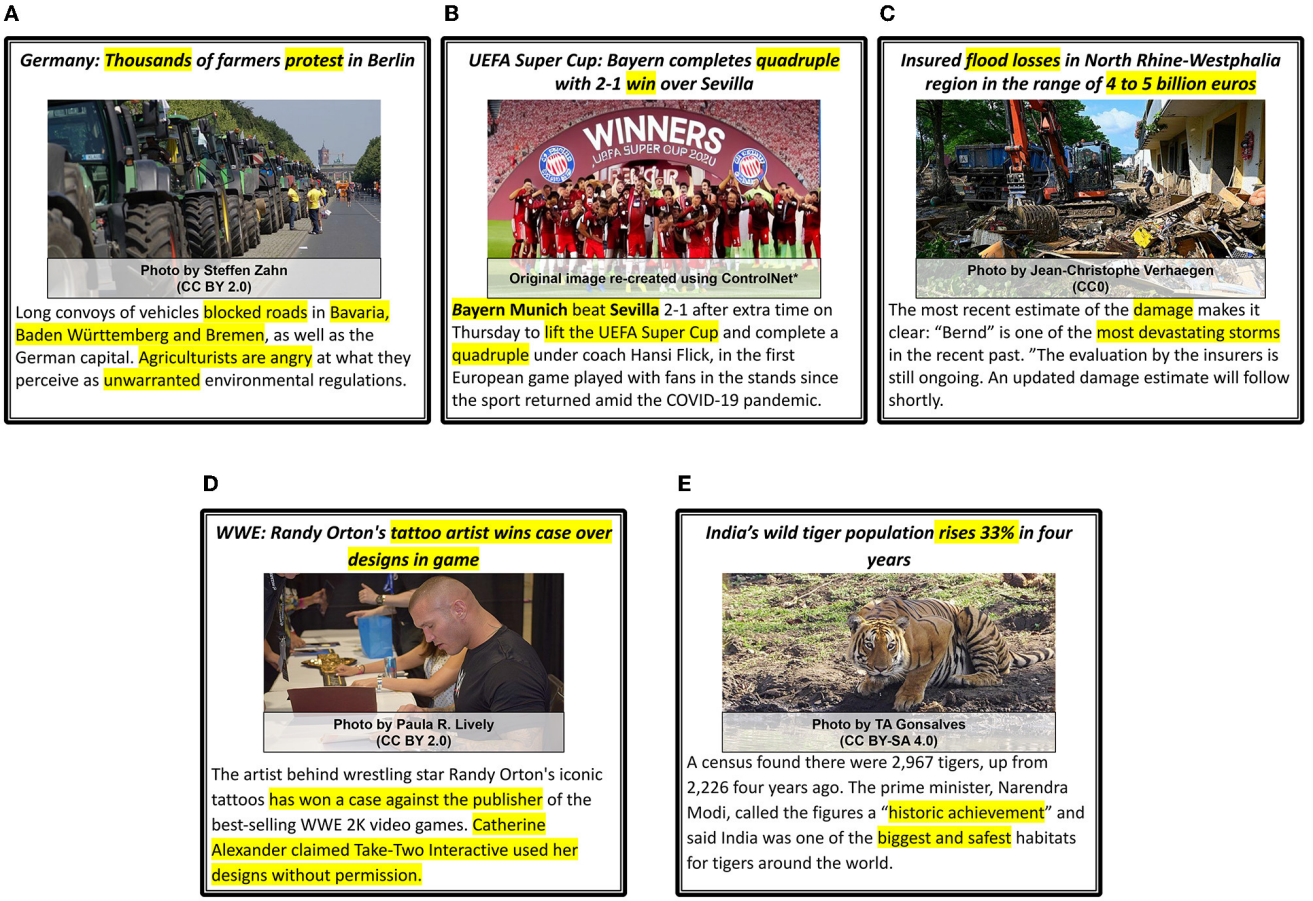
*Sentiment, Superlativeness* and *Impact* news values examples. All news values are described as **Category**, (textual or visual cues), *[Modalities: I (image), T (Text)]*. The segments in text that mention important words or phrases that indicate quantifiers, adjectives, mentions of disasters, etc. are highlighted (yellow). *Used ControlNet (Zhang and Agrawala, 2023) to re-create images because of licensing issues. **(A)** Sentiment : Negative, (Angry, unwarranted), [T], Superlativeness : Yes, (Thousands, tractors) [I,T], Impact : Medium, (protest, blocked roads in..) [I,T]. **(B)** Sentiment : Positive, (Win, celebration, happy), [I,T], Superlativeness : Yes, (multiple players, happy), [I], Impact : Medium, (win, celebration), [I,T]. **(C)** Sentiment : Negative, (losses, devastating), [I,T], Superlativeness : Yes, (heaps of trash, billion euros) [I,T], Impact : High, (damage, most devastating), [I,T]. **(D)** Sentiment : Positive (winning a case), [I,T], Superlativeness : No, [I,T], Impact : Low, (wins case, person vs. publisher), [T]. **(E)** Sentiment : Positive, (rises, safest), [T], Superlativeness : Yes, (biggest, safest), [T], Impact : High, (historic achievement), [T].

**Figure 12:**
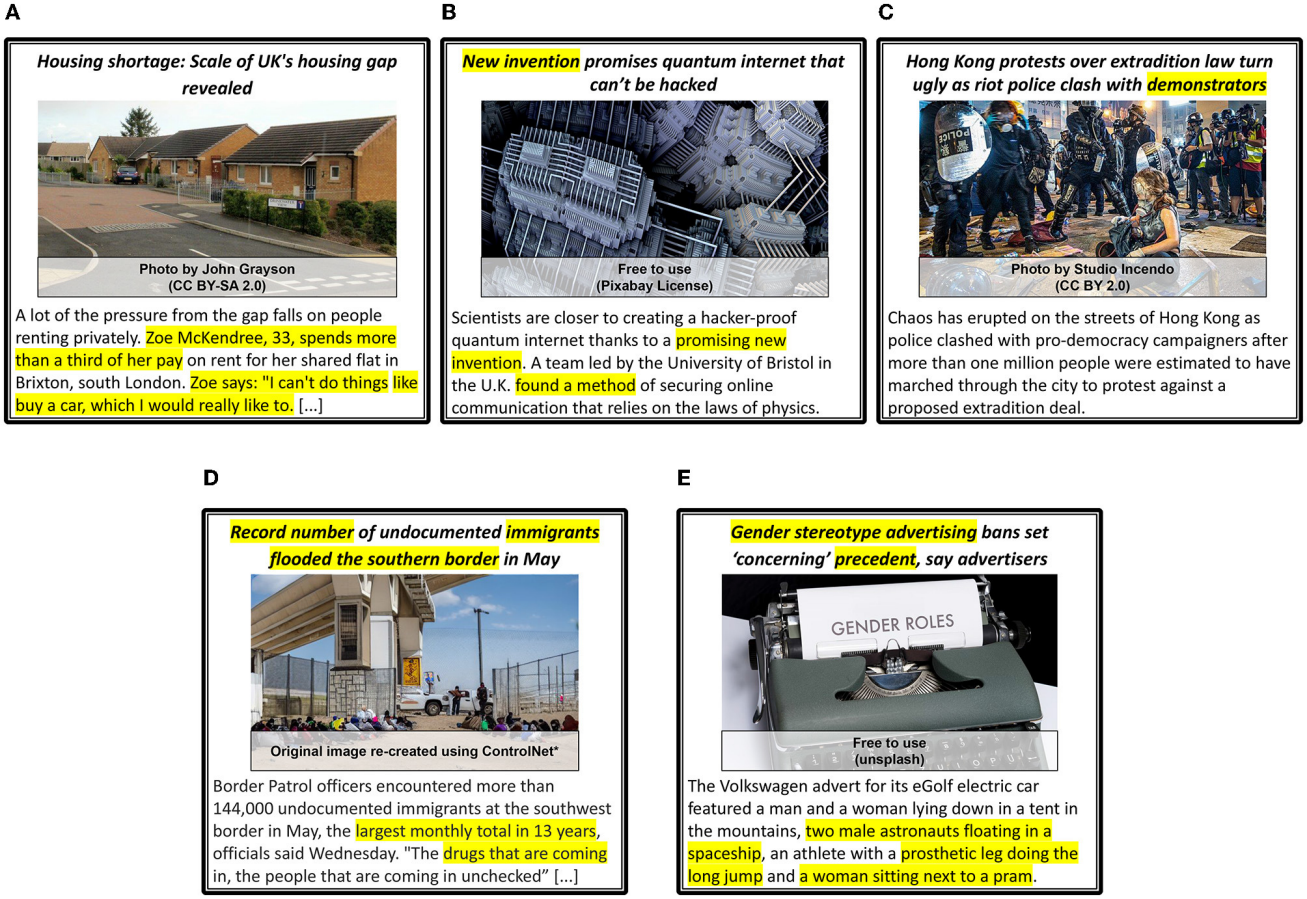
*Personalisation, Novelty* and *Consonance* news values examples. All news values are described as **Category**, (textual or visual cues), *[Modalities: I (Image), T (Text)]*. The segments in text that mention important words or phrases that indicate new or reoccurring events, mentions of disasters, etc. are highlighted (yellow). *Used ControlNet (Zhang and Agrawala, 2023) to re-create images because of licensing issues. **(A)** Personalisation : Yes, (Personal account), [T], Novelty : No, [I,T], Consonance : No, [I,T]. **(B)** Personalisation : No, [I,T], Novelty : Yes, (new invention) [T], Consonance : No, [I,T]. **(C)** Personalisation : Yes, (Focus on protestor), [I], Novelty : Yes, (contrast b/w police vs protestor) [I], Consonance : No, [I,T]. **(D)** Personalisation : No, [I,T], Novelty : Yes, (record number, police vs immigrants) [I,T], Consonance : Yes, (flooded, drugs, image usage) [I,T]. **(E)** Personalisation : No, [I,T], Novelty : Yes, (precedent), [T], Consonance : Yes, (News is about stereotypes), [T].

We provide five examples of news articles that are categorized with the respective *Proximity, Timeliness*, and *Eliteness* categories in Figure 10; five examples of news articles for which *Sentiment, Superlativeness*, and *Impact* news values are identified in Figure 11; and five examples of news articles that are categorized with *Personalization, Novelty*, and *Consonance* news values in Figure 12. Each sample is matched with a corresponding news value and its sub-category and marked with the modality (I: Image, T: Text) that provides the essential cues.

#### 3.3.1. Proximity

*Proximity* is defined by Caple and Bednarek (2016) as a *geographical or cultural nearness of an event or issue*. Based on this given definition, we divide it into three different components so that each can be analyzed separately. In addition to the geographical and cultural aspect of an event, we propose adding domain *proximity*, which indicates the topics covered in a news article.

- *Geographical proximity* is the distance between the event and the expected (target) audience. It can be seen as the scope of the event or the news, such that the news piece could directly or indirectly *impact* people who come under this scope. In addition to the individual locations mentioned in the article, we further sub-categorize this into four values which reflect the scope of the news article: *local* (a city), *regional* (multiple cities in the state/province), *national* (country or multiple states/cities in the country) and *global* (multiple countries). While it is relatively easy to identify locations and scope from text, geo-location estimation from images (Müller-Budack et al., 2018; Theiner et al., 2022) is a harder task that gives us the probable location(s) based on iconic landmarks, cultural symbols, artifacts, various other objects, including prominent people etc. Therefore, geographical *proximity* from images is limited to one (most probable) location unless there is text in the image, which helps to identify the scope. In Figure 10D, the news value of *Proximity* is categorized as *Geographical-National* since the text include entities such as *London, UK* and the image shows a well-known landmark in *London*.
- *Cultural proximity* is the reference to a particular religion, tradition, celebration, festival or ritual. Even though any group of people with some commonalities can constitute a culture, we adhere to the mentioned aspects only. Any other aspects that come under the broad definition of culture can be captured in domain *proximity* as topics in a news article. When words or phrases and visual cues in text and image respectively refer to one of these aspects, they are categorized as *culture-bound*; otherwise, they are *culture-free*. In example Figure 10C, the text includes the word “Christmas”, which makes the post *culture-bound*.
- *Domain proximity* refers to concepts or topics discussed in the news article discernible through image and text. In this case, concepts could be different in image and text, and a combination of both gives the list of all concepts. For example, broad topics such as business, technology, science, and sports are valid domains under which news articles are often organized in conventional newspapers and news websites. In addition, there can be other fine-grained topics based on events (such as Olympics) and themes in the news article. In all the examples in Figure 10, *Domain Proximity* lists topics extracted (manually) from both image and text.

#### 3.3.2. Timeliness

*Timeliness* is defined as the relevance of an event in terms of time of occurrence. We divide *timeliness* into four categories based on the date of publication and reference to exact time, date, or season in the news article: *old* (two years or before), *recent* (last two years), *ongoing* and *future*. As explained in Caple and Bednarek (2016), explicit verbal cues other than a date can be words like *today, yesterday* and verb tenses like *have been trying* in addition to the context around these phrases. For images, in the absence of text on the image, it is very challenging (Müller et al., 2017) to determine the date from a single image.

#### 3.3.3. Eliteness

*Eliteness* refers to the involvement of known individuals, organizations, or nations in an event. We consider only certain people and organizations under *eliteness* and not nations as defined in Caple (2013). Although, it can be true that certain developed countries get more coverage and be considered as elite in news value. However, considering certain countries as elite and others as not elite is reinforcing or introducing bias into the models that are (or will be) specifically designed to detect such attributes. As geographical *proximity* provides locations and scope of the event, the aspect of *eliteness* according to countries can be established by publisher-specific analyses based on *proximity*. People like celebrities, politicians, elite professionals (e.g., authors, scientists), and organizations that are well known to operate as for-profit or non-profit are considered of high status. From the text, named entities that have Wikipedia entries can be used to depict *eliteness*, and in the absence of all of these as *no eliteness*. In images, the same can be recognized via faces, context (microphones, cameras, police) and elements (such as a uniform) associated with an elite profession. As discussed in Caple (2013), *eliteness* can be identified via technical aspects such as camera angle with respect to the individual, where a low angle (viewed as looking up) can be hypothesized as suggesting the high status of the person in the image, although such attributions must always be treated with caution as they generally depend on other supporting accompanying cues. The example in Figure 10D has a news value *Eliteness* based on the following known persons mentioned in text: *David Beckham, James Bond, Mr. Bean* or *the Queen*.

#### 3.3.4. Sentiment

Sentiment refers to the negative and positive evaluation of events etc. in addition to language and vocabulary used in the news article. Although it is mentioned as *negativity* in journalism studies, we prefer the broader term *Sentiment* as both negative (e.g., disasters) and positive (e.g., winning a World Cup) events are covered as stories in the news. In the text, this can refer to certain events (like disasters, accidents, new invention, a peace treaty), and usage of positive vocabulary (tone/language) (e.g., happy, saved, beautiful) or negative vocabulary (e.g., terrible, bad, worried). In images, people with negative or positive emotions and effects of certain events mentioned before (e.g., football players celebrating a win, the aftermath of natural disasters) correspond to negative or positive *sentiment*. Similar to *eliteness*, technical aspects like high camera angle (viewed as looking down upon) with respect to the person of interest in the image may depict negative *sentiment*, as discussed in Caple (2013). The news value *Sentiment* is categorized into three classes, i.e., *positive, negative*, and *neutral*. For example, the sample in Figure 11E has a *positive sentiment* as the number of endangered species have increased. Whereas examples (a) and (c) have *negative sentiment*, because of the words like protest, angry, damage and devastating, and image in (c) showing damage and destruction.

#### 3.3.5. Superlativeness

*Superlativeness* refers to intensified or maximized aspects in the news article. The intensified aspects can be both in the positive (e.g., millions of dollars in profit) and negative (e.g., sharpest drop in the stock) direction, such that it is independent of the context. In the text, maximized aspects of the event can be identified via quantifiers (large numbers), intensifiers that emphasize amount, scale or size, and superlatives, all of which can be identified using the grammatical structure of text documents. It can also be established with metaphors and similes (like a raging river) that reflect the intensity or *impact* of the event. For images, *superlativeness* can be identified through repetition of key elements (e.g., soldiers marching, cars stuck in traffic), depiction of extreme emotions (anger, shock, fear) and placement of contrasting elements (e.g., a big new airplane standing next to a smaller one). *Superlativeness* can be classified into two categories, *yes* (presence of multiple identifiers) and *no* (absence of identifiers). For example, sample (e) in Figure 11 has *yes* as *superlativess* news value where the news article mentions it with words such as “safest” or “biggest”.

#### 3.3.6. Impact

*Impact* refers to positive or negative effects and consequences of the event covered in the news article. Although the *impact* is sometimes closely related to *superlativeness, impact* focuses more on the consequences of an event. In the text, *impact* can be identified via references to effects on individuals (e.g., unwarranted environment regulations on farmers, see Figure 11A), important and relevant consequences (e.g., the aftermath of a natural disaster, celebrations after a World Cup, see Figure 11C) and significance of the event (e.g., historic, momentous day). For images, similar to *superlativeness*, it can be identified through repetition (depicts the extent of *impact*) of certain elements and negative or positive (e.g., damage to public property, Olympic gold medal win) imagery of effects or the event. Like a few other news values, technical aspects in images like blurring because of excessive camera movement can also depict the event's *impact* (e.g., war, hostage situation) when viewed in combination with the text that describes the event. *Impact* is categorized into three classes, *low* [impacts number of individual(s)], *medium* (that affects a group of people in a community) and *high* (that affects larger regions or countries).

#### 3.3.7. Personalization

*Personalization* refers to personal aspects in the news article. *Personalization* is constructed when an abstract issue is made more personal via references to ordinary people, their emotions, experiences (e.g., eyewitness accounts) and stories in the news article. In the text, it can be identified by the context (e.g., detailed description of person's suffering or jubilation) and presence of a unknown person (if the mentioned person does not have an entry in Wikipedia). In images, visual cues like a close-up shot of an individual, focus on emotional response, facial expression, and singling out a person in a group of people (protester and police). *Personalization* is divided into *yes* and *no* categories. The sample in Figure 12C includes a *Personalization* news value because the image focuses on an ordinary person as a demonstrator. Text in sample (a) describes a personal account and situation of a person affected by high rent prices, realizing *personalization*.

#### 3.3.8. Novelty

*Novelty* refers to the new or unexpected aspects of the event. In the text, the aspect of *new* can be identified with evaluative words like *different, strange*, comparisons (with the past) that indicate unexpectedness like *never seen anything* and references to extreme emotions like shock and surprise. Unusual news or happenings that are categorized as bizarre news in media also construct *novelty*. For images, similar to superlativeness, extreme facial expressions indicating people being shocked or surprised and comparison between two or more elements to create a stark contrast in an image can also depict *novelty*. *Novelty* is divided into *yes* and *no* categories. The sample in Figure 12B has a *Novelty* news value because the text mentions the phrases “new invention”. Similarly the image in example Figure 12C realizes *novelty* by showing a contrast scene with one demonstrator surrounded by riot police.

#### 3.3.9. Consonance

*Consonance* refers to the expectedness (unlike *novelty*) of the event or presence of certain stereotypical aspects, which can be with respect to a person, community, organization or country in the news article. Just like *novelty*, expectedness in text can be identified with evaluative phrases like *famed for, notorious for* and comparisons like *yet another, once again*. Moreover, images can show aspects that fit a particular stereotype and combine with text to construct *consonance*. *Consonance* is divided into *yes* and *no* categories. The sample Figure 12D realizes *consonance* news value by associating certain traits (drugs, flooded the border) to immigrants that reinforce stereotypes.

### 3.4. Subjective interpretation

Recipients perceives information in the news differently depending on their background, knowledge and experience. This source of variation is far from arbitrary, however. With multiple presentation modalities such as image and text, different kinds of objective and semantic relations can be established between modalities from the available content and information, as explained in detail above. But, in addition, the differing sets of traits, such as *age, gender, education, reader's location, language, domains of interest, religion, click history, etc*., attributable to each recipient (reader) induce further changes in relations when contrasted with the content-based perspective. This change in objective and semantic relations, as well as some news values, is called *Subjective Interpretation*. One use case for this kind of analysis is reader-specific studies based on demographics and how the information composed using presentation modalities varies from the intended purpose. From a media studies perspective, given the cross-modal relations extracted from a news publisher's corpus and its reader base characteristics, one can analyse links between the content used for news vs the reader demographics. For instance, if certain image-text compositions are more prevalent for one type of readers' demographics or characteristics than the other.

The analysis can be further refined based on certain news values that are highly reader-centric and exhibit a different value from the reader's perspective. All news values except *timeliness* and *superlativeness* are constructed in the view of the target audience. For example, consider a news article about Germany winning a football match against France. The content would be mostly about how well Germany played, won the game, and construct the story with certain statistics about the game. Even though the article has a positive connotation, a French team supporter living in either Germany or France would probably perceive it differently from German football fans or even perceive it negatively. Here, we describe how news values may be varied from a recipient's perspective:

- *Proximity*: Based on the locations and places identified from the news article, geographical *proximity* for a recipient is categorized as *low* (near), *medium*, and *high* (far) based on geo-location and nationality of the reader. The sub-category for a reader can be computed using a threshold. For cultural *proximity, culture-bound*, and *culture-free* for a reader is based on its traits and cultural aspects (if any) and context in the news article. Lastly, domain *proximity* is based on the overlap of topics in the article and the reader's interests.
- *Sentiment*: Each recipient perceives the news and content in the article differently that can vary from the intended purpose of the article. In addition to polarity of the *sentiment* (*positive, negative and neutral*), it is further categorized into eight emotions (Mikels et al., 2005) as *fear, sadness, disgust, anger, amusement, contentment, awe*, and *excitement*.
- *Eliteness, impact, personalization, novelty, consonance*: All other news values can be different based on the recipients' traits and are sub-categorized as *yes* and *no*. For instance, a prominent individual or an organization mentioned in the article might not be relevant and not considered elite by the reader because of a different location and interests. *Impact*, in this case, refers to whether the reader is affected by the event or its effects and consequences. As *personalization* is the “human” aspect in the article, it refers to whether a recipient relates to the personal aspects or not (e.g., photo of a person crying in front of a destroyed building after Earthquake). Lastly, whether a reader considers the events as expected or unexpected is covered in *consonance* and *novelty*, respectively.

From the computational perspective, we have three kinds of resources here, first is cross-modal relations or news values based on content, second are reader characteristics, and lastly the reader-centric news values. Treating two as inputs and one as the output in a predictive task, we can consider these problems under user modeling to predict readers' preferences, readers' characteristics or improvise information dissemination (language and image usage).

### 3.5. Interplay between different aspects

In this section, we discuss some illustrative articles from different news media channels. We particularly pay attention to author intent, cross-modal relations and news values in order to show the necessity of adequately reflecting the sometimes quite complex interplay between these different aspects. In the previous sections, we presented examples of each aspect with pairs of image-short text (headline or caption). Here, we extend the focus to include an article's components, including the headline, image captions and some parts of the main text. Structural analysis of this kind has also been performed and shown specifically for news values by Caple and Bednarek (2017), enabling the comparison and interaction of news values across an entire article. Now, we show how it is also beneficial to perform analyses that incorporate our broader set of perspectives that go beyond news values alone.

**Figure 13:**
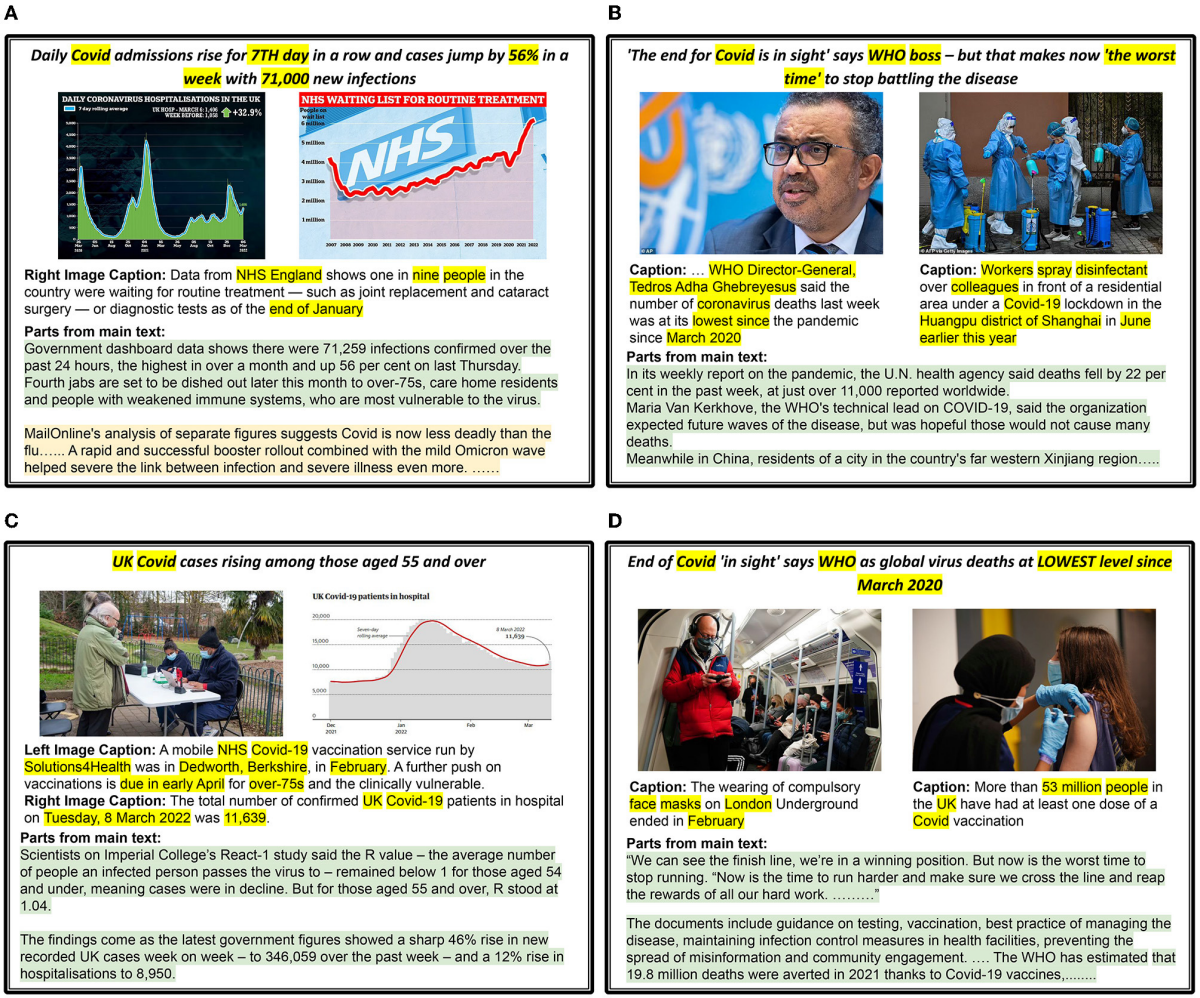
Examples of news articles on COVID-19 with multiple images, captions and parts from the main text. Left and right column news are from 10^*th*^ March and 15^*th*^ September 2022, respectively. Yellow highlighted text - refers to entities/concepts and news values clues. Green highlighted text - statements being informative (reporting facts or opinions of experts) and orange highlighted text - statements being persuasive (author's own analysis/opinions). **(A, B)** Daily mail. **(C)** The guardian. **(D)** Daily mirror.

In Figure 13, we present four articles for comparison, all related to COVID-19 and taken from top-read daily news media channels in the United Kingdom. In the top row (Figure 13A), we see two articles from *dailymail.co.uk* (a) and (b); bottom left an article from the *guardian.com* (Figure 13C); and bottom right an article from the *mirror.co.uk* (Figure 13D). The articles in each column are drawn from the same dates in March and September 2022, respectively. The left column articles are about the “*rise in weekly COVID-19 cases by 50%”*, and the right column articles are about “*WHO's announcement or statement about the end of the COVID-19 pandemic”*. In the following, we discuss differences and similarities across the three media channels drawing on the perspectives defined in our analytic framework.

Starting with the two *Daily Mail* articles, a common observation is the use of images and text to provide both a supporting and a contrasting viewpoint with respect to the headline. In the left article,^2^ several graph images (three with black background, one shown in Figure 13A) are used without direct references in the text and no captions (*Uncorrelated OO and no extension*). Here, the text comes to the same conclusion (*Equal STATUS* and there is *no modification*) evident in the image (*high-positive SC*). These three images and the supporting texts followed by the author's own opinions (sometimes referring to theories behind the increase without citing sources) promote (*persuasive author intent*) the main theme of “rise of infections”. Conversely, three more graph images are included in the article to provide a different viewpoint on “covid being less deadly than the flu” and “covid impact on rise in the waitlist of routine treatments”. One of these images (shown in Figure 13A) has a caption which refers (*Image STATUS*) to the image (has a common entity “NHS”, *partial OO*), summarizes the graph (*high-positive SC*), and adds more entities (*Text extends Image*). The image, on the other hand, amplifies (*amplification*) the “one in nine people waiting” messaging by showing the difference in people on the waitlist now vs. pre-covid times. Interestingly, this image caption is not discussed and referenced anywhere in the main article but instead in a subordinated text fragment embedded within the article, which reflects the complex structure used by the *Daily Mail*. In addition, another image (not shown in Figure 13A), which also has a caption, exhibits similar image-text relations, presenting in detail opinions from the author and quoted excerpts from cited experts (*informative, reports facts, and opinions*). In summary, the majority of the article has image-text pairs that exhibit different relations as in the beginning of the article, and promotes (*persuasive author intent*) the “living with covid” perspective, in contrast to the headline (*demotes, persuasive author intent*). This demonstrates clearly how important it is that any analysis is capable both of differentiating finer structural units between which relations can hold and of allowing those relations to be divergent.

The article also constructs distinct news values of *superlativeness* (large numbers, rise of cases and waitlist), *impact* (infection rise, deaths, routine treatments), *proximity* (national-UK and international-European), *timeliness* (recent, ongoing, and old), *sentiment* (positive and negative), and *eliteness* (NHS, other organizations, experts). As with the image-text relations, here there is variation over the extent of the article of news values as well as between the image and text. For instance, the main article text constructs the news value of *timeliness* as *ongoing*, whereas images explicitly construct *recent and old* by showing years as far back as 2007. Similarly, *superlativeness* (from thousands of cases to millions of people on the waiting list), *impact* and *sentiment* (from *negative*-rise in cases to *positive*-covid less deadly than flu to again *negative*-rise in wait list) are constructed in different ways across the extent of the article.

In a similar manner, the article^3^ on the right, first, primarily reports (*informative, facts and opinions*) regarding the statement given by WHO and, later, provides a contrasting viewpoint on the long period lockdowns in China and their impact on residents. Two out of three images (one shown on the left of Figure 13B) are stock photographs (*partial OO* and *low-positive SC* and *no modification*) with captions providing the main message (*Text STATUS*) and additional entities (*Text extends Image*). A notable difference from the other *Daily Mail* article is the absence of the connection to the rest of the main article text, except for the news value of *eliteness* via images. This could be because the first article is written by a health and science editor for an in-depth analysis. The third image (shown on right in Figure 13B) depicts the situation in the caption (*high OO* and *high-positive SC*) and exhibits the same relations as the previous two image-caption pairs. In addition, the caption here exhibits the different news value of *timeliness* (June) in comparison to the lockdown news (from September) from China. Furthermore, similar to the other article, the later text on the lockdown in China consists of the author's claims and opinions without concrete source references or quotes from experts. In summary, several distinct news values are constructed “simultaneously” in the article: *eliteness* via both image and text (Director-General and WHO), *novelty* (lowest figures) via text, *international proximity* (U.S., England, China, World) in text, positive *high impact* (lowest figures, end of pandemic) and negative *medium impact* (resident's condition in a Chinese city), and lastly *sentiment* (from *positive*-lowest cases worldwide to *negative*-hunger, forced quarantines in China).

In contrast to the *Daily Mail*'s article on the left, *The Guardian*^4^ article (also written by a science editor) covers the news on the rise in cases and its impact on people aged 55 and above. The article focuses on facts from government reports and figures, scientific reports and opinions of five different experts (*informative, facts, and opinions*). Moreover, the structure of the text (paragraphs instead of sentences) and the use of images is quite different from the *Daily Mail*'s article. For example, the left image is not referenced in the main text, but the image-caption pair is relevant for the construction of news values of *personalization* and *impact*, resulting in *high-positive SC but no modification* with the caption and some of the text snippets in the main text. In addition, the image-caption pair has a *partial OO* and *both extend* in terms of new information, with the text as the main message *(Text STATUS)* and constructing news value of *local proximity* (Dedworth, Berkshire), *timeliness* as ongoing and future, and *eliteness* (NHS, NHS personnel). Conversely, the second graph image from *gov.uk* acts as a placeholder image (in this case, of COVID-19 patients in hospitals) and is not referenced in the main text; it is then only weakly linked (*low-positive SC*) to show the rise in cases and hospitalizations (*quality modification*, not matching the figures). Such image-text relations consequently suggest a sub-optimal combination of image and text in terms of information dissemination. In addition, several other news values are constructed in the rest of the text: *sentiment* as mostly *negative, superlativeness* with large numbers, *high negative impact* for the nation's elderly, *local and national proximity* (Berkshire and England), *timeliness* as *recent, ongoing, and future*, and *eliteness* (institutes, experts, government, and NHS).

Lastly, the *Daily Mirror*^5^ article shows the most contrasting use of images with respect to the article and the main text. In three (two shown in Figure 13D) out of five images in the article, the news value of *personalization* (focus on laypersons) is constructed. The article's main text stands in contrast to the *Daily Mail* article in that most of the focus is on disseminating the WHO's announcement and information from documents shared by the WHO (*informative, facts, and opinions*). Moreover, the rest of the text only points to vaccine strength, decreasing the number of cases, and the alert levels in the United Kingdom. In contrast, two image-caption pairs (one shown on the left in Figure 13D) exhibit *negative SC* and *quality modification* (image showing people wearing masks vs. mask mandate ending in February in text). In terms of objective relations, there is *partial OO* and *Image extends Text*. This could be because of either the use of older dated image or to signify that people are still cautious in public places, and so serves to persuade the reader to be cautious (*persuasive*, promote the “cautious perspective” or demote the “end of covid” message). In addition, the news value of *superlativeness* is constructed with multiple people in the image to emphasize this cautiousness aspect. This is a notable difference between the *Daily Mail* and the *Daily Mirror* articles: both are *informative* and *persuasive*, but compose their articles via different usages of text, image-caption pairs and news values. While the *Daily Mail* relies on expanding proximity (to China's condition) and *high-positive SC* image-text pairs, the *Daily Mirror* shows *negative SC* image-text pairs and news value of *personalization* to contrast with the central theme of the article.

## 4. Discussion and conclusions

In this final section, we briefly discuss the computational feasibility of different parts of the framework. Moreover, we outline use cases and applications of the framework that can be interesting directions of research in computational modeling, multimodal analytics and social science and then briefly conclude.

### 4.1. Feasibility of computational approaches

***Author intent***: Previous work related to author intent tackles the problem of identifying news (reporting of factual information) vs. opinion (Krüger et al., 2017), the persuasive effect of news editorials (Baff et al., 2020) and argumentation strategies in news (Khatib et al., 2017). These earlier approaches rely on engineered lexical features which are not robust against changes in topics and not generalisable to unseen (during training) news publishers. Recently, Alhindi et al. (2020) combine argumentation features and computational language models like BERT^6^ (Devlin et al., 2019) to identify news vs. opinion. They show promising performance in comparison to previous approaches across datasets and publishers. For multimodal author intent detection, Kruk et al. (2019) benchmark several large multimodal model features to classify author intent from Instagram posts. The recent work suggests that a combination of unique lexical and multimodal features can be used to classify author intent classes as described in our framework.

***Cross-modal relations***: There is a wide body of work on vision-language modeling (Gan et al., 2022), where the goal is to learn visual and multimodal features from a large-scale corpus of image-text pairs from the Web. These models often have deep network backbones like BERT (Devlin et al., 2019) and are trained with loss functions such as masked language modeling, masked image region prediction and image-text matching. Recent work on image-text matching and retrieval (Cao et al., 2022) has explicitly focused on refining cross-attention mechanism and local alignment to get better retrieval performance. Consequently, these models focus more toward aligning literal (objective relations) relations than abstract and high-level semantic relations (semantic correlation STATUS, modification). Recent work (Xu et al., 2022) suggests that weakly aligned (e.g., low-positive SC) and unaligned (e.g., zero, negative SC) pre-training can be beneficial and is still understudied. On the contrary, recent work such as Henning and Ewerth (2018) and Otto et al. (2020) have tailored multimodal neural networks in order to predict relationship metrics like CMI, SC, and STATUS. Müller-Budack et al. (2021b) have taken a different approach of predicting image-text consistency in news over named entities like persons, locations and events, using several supervised pre-trained model features for face recognition, geo-location estimation and event detection.

***News values***: For all the news values except *sentiment*, parts of speech tagging (Chiche and Yitagesu, 2022) and named entity recognition and linking (Luo et al., 2015) can identify words and phrases for each news value. With the identified words, one can either create a rule-based classifier to classify the news values or train a data-driven classifier. In addition, temporal reasoning and extracting temporal and causal relations can be beneficial to identify the impact and novelty of the event in news (Caselli and Vossen, 2017). Both lexical and neural network-based classifiers are well explored for sentiment and can be used to detect sentiment in news (Mello et al., 2022). Moreover, convolutional neural networks (CNN) and multimodal neural networks can also be applied for visual (Ortis et al., 2020) and multimodal (Soleymani et al., 2017) sentiment analysis. Similarly, topic modeling can be performed using recent state-of-the-art approaches like BERTopic (Grootendorst, 2022), which combines word embeddings with TF-IDF (Term Frequency-Inverse Document Frequency) to create topic representations. Recently, some works have explored deep networks for image understanding in combination with text-based topic detection for news videos (Li et al., 2017) and Twitter posts (Zhang et al., 2019). For images, while there is ongoing research for tasks related to some news values, others, like stereotype identification and novelty detection, are still yet to be explored. For other news values, such as identifying location, visual cultural symbols and identifying objects for more context, open vocabulary multimodal models like CLIP (Contrastive Language Image Pre-training) (Radford et al., 2021) can be used to detect the presence of things of interest. Some news values require face recognition (Zhu et al., 2021) and facial analysis (Li and Deng, 2022), which can be efficiently done with the use of CNN-based face detection and facial expression prediction. For identifying the impact of events in images, deep networks can be applied for a disaster impact assessment on aerial imagery (Gupta et al., 2021) and image-text pairs of tweets (Rizk et al., 2019). Lastly, there are a few works where a BERT-based model has been used for detecting racist (Fokkens et al., 2018), gender (Chiril et al., 2021) and immigrant (Sánchez-Junquera et al., 2021) stereotypes in news, tweets and political debates respectively. As each news value is a separate task, an ensemble or a rule-based model using different features and labels extracted from the discussed models could also be explored in a multi-task and multimodal learning setting.

### 4.2. Applications and use cases

As the computational modeling of cross-modal relations is an active research area (see Section 2.2), both the objective and the semantic relations are interesting. For example, they can serve as the basis for creating novel datasets for news analysis for multimodal learning tasks to foster further research in the area. Recently, there have been attempts to create datasets for non-literal relations between image and text in advertisements [e.g., Zhang et al. (2018)], tweets [e.g., Vempala and Preotiuc-Pietro (2019)], Instagram posts (Kruk et al., 2019), and across domains (Otto et al., 2019b). However, only Otto et al. (2019b) decouple relations at conceptual and meaning level motivated from semiotics and none of them are in the news domain. Recent work by Alikhani et al. (2020), Sosea et al. (2021), and Utescher and Zarrieß (2021) have posed new research directions and interesting applications of modeling a variety of image-text relations. From the perspective of news values, recent work on automatic extraction of news values from news text (di Buono et al., 2017; Piotrkowicz et al., 2017; Belyaeva et al., 2018) can be extended to both images and text with novel datasets and multimodal modeling contributions. The challenge of detecting news values in both image and text can be at the level of just predicting the type and class of news value to detecting spans of text and regions in the image signifying the news value. From the user modeling perspective, there has been much work on news recommendation based on Twitter user profiles (Abel et al., 2011, 2013), user graphs (Wu et al., 2021), click patterns and behaviors (Wu et al., 2019, 2020) and multimodality (Wu et al., 2022b). Wu et al. (2022a) present a detailed survey of personalized news recommendation and organize relevant literature by the type of news content, properties (like a publisher), dynamic information (like popularity, click-through rate) and user features (like user networks). Our framework includes the recipients' perspectives in the news process and provides a combined view with news values where contributions can be made from both the content and user modeling for further research in the area.

Modeling semiotic relations at the conceptual and meaning level for news can be important for news-specific downstream tasks like the detection of out-of-context images (Aneja et al., 2021; Luo et al., 2021) or fake news (Giachanou et al., 2020; Singh and Sharma, 2021). Both applications can benefit from predicting additional attributes relevant to news dissemination when coupled with news values from a multimodal perspective. For instance, Springstein et al. (2021) present an application where cross-modal consistency between text and photo is checked for entity types persons, locations and events. Additional attributes like news values can be added as consistency parameters to enrich existing models. Accurate identification of news values in text and images can also help estimate the newsworthiness of informal news on social media. As mentioned before, the news recommendation system is an interesting application that can be enriched with predictable news values from the content combined with available user traits and click patterns. News retrieval (Tahmasebzadeh et al., 2021) can be enriched based on image-text relations and news values. For instance, news can be retrieved and ranked higher of a particular event where images are relevant (*high objective overlap and semantic correlation*) to the news text and includes personal stories or eyewitness accounts (*personalization*).

From the social science perspective, some of the models and applications discussed above enable news analysis, user studies and news comparisons. For instance, the automatic identification of news values in text and image can aid in studying narrative differences between mainstream news and news on social/alternative media. In terms of news comparison and supporting discourse analysis, Pollak et al. (2011) use text mining to find contrasting patterns between coverage of “2007 Kenyan elections and post-election crisis” in local (Kenyan) and Western (British and US) newspapers. As presented in the framework, fine-grained relations between image and text coupled with news values can provide a multimodal perspective of image and text usage in important events (like an election crisis) across news publishing platforms. Recently, O'Halloran et al. (2021) have introduced a platform called Multimodal Analysis Platform (MAP) for searching, storing and analysing multimodal content (text, images, and videos) in online social and news media. However, the tool focuses on extracting information from individual modalities and so far does not integrate this as would be necessary to operationalize meaningful search and descriptive analysis of meanings emerging from combinations of modalities. The integration of analytical capabilities of image-text relations and news values would therefore be an important additions for such a tool in order to perform analyses going beyond the descriptive aspects.

### 4.3. Conclusions

In this paper, we have reviewed multimodal semiotics and computational science literature to derive a framework of image-text relations, news values, and author intent for the support of richer multimodal news analysis. The presented framework covers the entire spectrum of the news process, from news production to news consumption. Taking inspiration from semiotics as well as recent work in the fields of multimedia and multimodal machine learning on relations between image and text, we outlined a framework based on objective information (*objective relations*), meaning formation (*semantic relations*) and relative relevance (*STATUS*) between the two presentation modalities. We also introduced a new relation called *modification* as a sub-category of semantic relations better to capture the role of images in news dissemination. Further, for news-specific analysis, we introduced the aspect of *author intent* to identify the author's primary purpose behind an article and *news values*. While there have been a few attempts at automatically detecting some news values from text and particularly news headlines, image and text together have yet to be considered in computational research, so this is an upcoming specific research challenge. Finally, we included *subjective interpretation*, which captures the end user's and reader's subjectivity toward presentation modalities and news value aspects. Relevant examples for each part of the framework and comprehensive comparisons with existing work were also presented. Finally, we concluded the paper with possible research directions, applications and relevance of the work from a social science perspective.

## Data availability statement

The original contributions presented in the study are included in the article/supplementary material, further inquiries can be directed to the corresponding author.

## Author contributions

GC devised the project, developed main ideas, prepared examples, theory and review, and took lead in writing the manuscript. SH, EM-B, and CO provided feedback and fruitful discussions, edited parts of the manuscript, and helped shaping the research and ideas. JB and RE provided critical feedback, helped in shaping the manuscript and bring out contributions, and edited the manuscript. All authors contributed to the article and approved the submitted version.

## Footnotes

^1^ https://theconversation.com/3-2-billion-images-and-720-000-hours-of-video-are-shared-online-daily-can-you-sort-real-from-fake-148630

^2^ https://www.dailymail.co.uk/news/article-10599205/Daily-Covid-admissions-rise-7TH-day-row-cases-jump-56-week.html

^3^ https://www.dailymail.co.uk/news/article-11213749/WHO-boss-says-end-Covid-sight-says-means-worst-time-up.html

^4^ https://www.theguardian.com/world/2022/mar/10/uk-covid-cases-rising-among-those-aged-55-and-over

^5^ https://www.mirror.co.uk/news/world-news/end-covid-in-sight-says-27996521

^6^Bidirectional encoder representations from transformers.
